# Graded Calorie Restriction Causes Graded Slowing of Epigenetic Ageing in Mice

**DOI:** 10.1111/acel.70342

**Published:** 2025-12-23

**Authors:** Timothy P. Moulds, Sharon E. Mitchell, Xiaojing Yang, Wei Guo, Emily Chen, Peter D. Adams, John R. Speakman

**Affiliations:** ^1^ School of Biological Sciences University of Aberdeen Aberdeen Scotland; ^2^ Zymo Research Irvine California USA; ^3^ Sanford Burnham Prebys La Jolla California USA; ^4^ Shenzhen Key Laboratory of Metabolic Health, Center for Energy Metabolism and Reproduction Shenzhen Institutes of Advanced Technology, Chinese Academy of Sciences, and Shenzhen University of Advanced Technology Shenzhen China; ^5^ State Key Laboratory of Molecular Developmental Biology Institute of Genetics and Developmental Biology, Chinese Academy of Sciences Beijing China; ^6^ Institute of Health Sciences China Medical University Shenyang Liaoning China

**Keywords:** ageing, calorie restriction, DNA methylation

## Abstract

DNA methylation variation is associated with chronological ageing. Calorie restriction (CR) prolongs lifespan and healthspan in many species. Our hypothesis is that CR has an impact on DNA methylation patterns with increased CR leading to slower epigenetic ageing. We studied the effects of graded CR in male C57BL/6J mice on liver DNA methylation. Mice were fed *ad libitum* (AL) in the dark‐phase or restricted by 10%, 20%, 30% or 40% from 5‐months old for 19‐months. Livers were collected in surviving mice at 24‐months old and DNA methylation measured. Comparisons were made to 8‐month‐old AL fed mice. DNA methylation was significantly related to graded CR in a subset of cytosine‐guanine dinucleotide (CpG) sites. In a substantially similar subset of CpG sites, DNA methylation in 24‐month‐old mice fed 40CR moved towards the values in 8‐month‐old AL fed mice, resulting in an average effective epigenetic age of about 12‐months, indicative of slower epigenetic ageing. DNA methylation at several CpG sites was sensitive to glucose intolerance and circulating insulin levels, consistent with the impact of this nutrient sensing pathway on ageing. We focussed on genes where multiple CpG sites were significant for DNA methylation change with CR and found many have been implicated in age‐associated liver diseases. In summary, the benefits of CR include modification of epigenetic signatures in the direction of slower ageing, consistent with the life extending effects of CR. Whether this effect is causal for the life extension under CR, and the mechanism by which it occurs remain unanswered questions.

## Graphical abstract

Graded calorie restriction in male mice modified epigenetic signatures of the liver and slowed ageing rate in a graded manner.

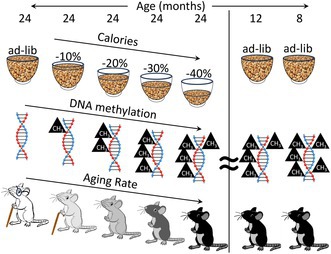

## 1 Introduction

The global population aged 65+ years is growing at about 30 million per year, leading to major societal challenges (United Nations 2023). During ageing, molecular damage accumulates over time, leading to reduced homeostasis and increased susceptibility to age‐related disease (Tenchov et al. 2024). Ageing is heterogeneous, complex, and multi‐factorial (López‐Otín et al. 2013). There is a gap of about 10 years between total and healthy lifespan, and this gap is increasing (World Health Organisation 2019). The risk of diseases including cancer, neurodegenerative disorders, cardiovascular disease, and diabetes increases as we age, leading to great concerns as the population of aged individuals rises (Li et al. 2021).

Calorie restriction (CR) extends both health and lifespan in many species (Green et al. 2022; Speakman and Mitchell 2011). Across rodent studies, lifespan extension due to CR was linearly related to the extent of restriction, up to at least 65% restriction (Speakman et al. 2016). These particular mice had increased survival compared with mice fed *ad libitum* (12AL) in the dark‐phase by 14%, 31% and 48% when restricted by 20%, 30% or 40% of their 12AL intake (Mitchell et al. 2023). While our results are in agreement with recent work showing lifespan extension was proportional to the level of CR and intermittent fasting (Di Francesco et al. 2024), others have reported the benefits of CR peaked at around 20% restriction and was not extended in a graded manner at 40% CR (Mitchell, Madrigal‐Matute, et al. 2016). Over a series of previous publications, we have reported numerous responses in 8‐month‐old mice with final 3‐month graded CR including profound changes in gene expression across multiple tissues (Derous et al. 2016, 2017; Mitchell, Delville, Konstantopedos, Derous, et al. 2015; Mitchell, Delville, Konstantopedos, Hurst, et al. 2015; Mitchell, Tang, Kerbois, Delville, et al. 2015; Mitchell et al. 2017; Mitchell, Delville, et al. 2016).

DNA methylation (DNAm) involves the covalent attachment of a methyl group to the fifth carbon atom of the cytosine residue at a cytosine‐guanine dinucleotide (CpG) (Jones et al. 2015). About half of the CpG sites in mammalian genomes are found in the promoter regions of genes (Jones and Baylin 2002), and increases in DNAm may silence gene promoters (Ehrlich 2019). It is contended that DNAm alterations across multiple CpG sites may be functionally more important than alterations at a single site (Lövkvist et al. 2016). The exact relationship between the presence of, and changes to, DNAm and gene expression is complex (Horvath and Raj 2018). Nevertheless, damage to the human DNAm landscape is associated with many age‐related diseases (Seale et al. 2022), and DNAm changes may underpin the gene expression changes observed in response to CR.

Epigenetic clocks sample DNAm at several hundred or more CpG sites to develop an epigenetic age that is calibrated to chronological age (Horvath and Raj 2018; Stubbs et al. 2017). Results here employ the epigenetic clock DNAge (Zymo Research) developed for mice (Coninx et al. 2020). CR modifies DNAm and CR has been shown to delay age‐related DNAm alterations across several species including humans and mice (Maegawa et al. 2017). CR reduces the pace of biological ageing, based on a DNAm biomarker, but did not significantly alter biological age estimates from several epigenetic clocks for humans in the Comprehensive Assessment of Long‐term Effects of Reducing Intake of Energy (CALERIE) randomised controlled trial (Belsky et al. 2022; Waziry et al. 2023). However, a principal component‐based clock found significant reduction in biological age in the same CALERIE data (Fong et al. 2024). A previous study of a small sample of mice suggested there was some reduction in epigenetic age by CR (Wang et al. 2017). Here we characterised the changes in DNAm in 24‐month‐old male C57BL/6J mice in response to graded levels (10%, 20%, 30% and 40%) of long‐term (19‐months) CR, and compared these to 8‐month‐old 12AL mice. We thereby investigated whether CR‐related changes in DNAm cause slowing of epigenetic ageing.

## 2 Results

Morphological and physiological measurements were made on 33, 24‐month‐old male C57BL/6J mice (Mitchell et al. 2023), with each exposed to one of five CR levels: 12AL, 10CR, 20CR, 30CR and 40CR for the previous 19 months (Supporting Information Table S1). The variables measured were cull body mass; basal metabolic rate (BMR); physical activity (PA), body temperature (Tb), food anticipatory activity (FAA); circulating leptin, insulin, and insulin like growth factor‐1 (IGF); catalase and superoxide dismutase (SOD) antioxidant activity and DNA damage in the liver, fasting glucose (Fg) and the area under the Glucose Tolerance Test curve (GTT). Similar measurements were obtained for 8‐month‐old 12AL mice which acted as a reference point for epigenetic age analysis. The measurements on 24‐month‐old mice served as predictor variables in linear regression analyses of factors linked to DNAm. All data morphological, physiological and DNAm measurements and whether the CpG site is within a promoter region and whether shore/island/open sea etc. will be uploaded to our Open Science Framework for ‘The graded Calorie Restriction project’, https://doi.org/10.17605/OSF.IO/9YATH.

### 2.1 CpG Sites With Significant Variation in DNA Methylation

**FIGURE 1:**
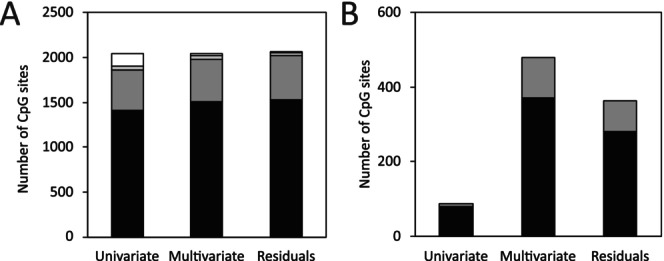
Disposition of the 2045 CpG sites at which DNAm was measured for each suite of linear regression. (A) the number of retained intragenic CpG sites (black), retained CpG sites in intergenic regions (dark grey), CpG sites excluded after removal of outliers (light grey) and excluded for non‐normal residual values (white). (B) The number of intragenic CpG sites (black) and CpG sites in intergenic regions (dark grey) that were statistically significant for variation in DNAm for each suite of linear regression.

DNAm values were obtained from liver samples at the same 2045 CpG sites for each mouse. All 2045 CpG sites in our analysis were identified as exhibiting age‐associated DNAm changes during the development of the DNAge epigenetic clock (Coninx et al. 2020). There were 1556 intragenic CpG sites within 214 genes and 489 intergenic CpG sites. We refer to genes by symbol, with the full gene names from Mouse Genomics Informatics (Baldarelli et al. 2024) presented in Supporting Information Table S2. Three linear regression models were used to identify CpG sites where there was a strong relationship between change in DNAm and change in potential predictor variables. DNAm at each of the 2045 CpG sites was evaluated individually by each suite of regression models. The first approach involved univariate regression for DNAm variation with CR. Next, to identify additional variables that impact DNAm, we carried out multiple regression of DNAm against all possible combinations of 14 predictor variables in Supporting Information Table S1 (16,383 different regression models at each CpG site). The third approach also involved multiple regression and consisted of regressing DNAm against all combinations of predictor variable residual values after removing the effect of CR from both the predictor variables and DNAm; this approach alleviates concerns of collinearity between CR and other predictor variables and removes the effect of CR on DNAm. CpG sites and the number of genes retained in the analysis after eliminating sites for outliers and non‐normal residuals are summarised in Figure 1, as are the number of significant CpG sites for each regression model.

In assessing the strength of the relationships between predictor variable(s) and DNAm we did not differentiate between DNAm increasing or decreasing with the predictor variable(s). Greater methylation of CpG sites in a gene promoter region has been associated with silencing gene expression, although recent work has illuminated a complex relationship between DNAm change and gene expression that involves other factors such as chromatin state information (Seale et al. 2022; Teschendorff and Relton 2018). For each of the multiple regression models, at each CpG site, the impact of predictor variables on DNAm variation was identified on the basis of the Akaike Information Criterion (AIC) value (Akaike et al. 1973). We term the model with the lowest AIC value to be the optimum model for that CpG site in that suite of linear regressions.

To determine statistical significance, we ranked the linear regression *F*‐statistic *p*‐values of the optimum models for the two multiple regression approaches and the univariate models at all CpG sites. After accounting for multiple statistical tests using the Benjamini‐Hochberg procedure, we determined those CpG sites at which DNAm variation with predictor variable(s) was significant (FDR‐adjusted *p*‐value < 0.05) (Table 1). We further assessed the importance of each predictor variable within the multiple linear regression results by ranking the *t*‐test *p*‐values of all variables in the optimum models and, again accounting for multiple testing, we identified significant (FDR‐adjusted *p*‐value < 0.05) predictor variables at each CpG site.

**TABLE 1:** Significant intragenic CpG sites in relation to CR and other predictor variables using (a) Linear regression of DNA methylation against CR, (b) multiple linear regression against all measured variables and (c) multiple linear regression after accounting for CR, that is, regression against residuals.

Gene (no. CpG sites sampled)	Linear Regression			Gene (no. CpG sites sampled)	Linear Regression			Gene (no. CpG sites sampled)	Linear Regression		
	Univariate	Multiple	Residuals		Univariate	Multiple	Residuals		Univariate	Multiple	Residuals
1010001N08Rik(14)	0	8 CR B Ca	10 B Ca	Gm10463(5)	0	1	0	Nlrp6(4)	3 CR	4	0
1700001O22Rik(10)	0	4	1	Gm12446(9)	0	1	1	Nt5c1a(8)	1	2	2
2700033N17Rik(4)	0	1	1	Gm12688(13)	0	3	1	Ntng2(6)	0	0	1
2900026A02Rik(5)	0	3	1	Gm14207(9)	0	2	2	Numa1(6)	0	1	1
9130019P16Rik(4)	0	0	1	Gm19810(11)	0	3	3	Osbpl7(7)	0	1	1
AC165157.2(15)	0	1	1	Gm20388(6)	1	1	0	Padi4(7)	0	1	1
Actl7b(9)	3 CR	2	0	Gm2093(3)	0	1	1	Pde4dip(10)	1	3	2
Adamtsl5(7)	1	4	2	Gm21297(20)	0	1	1	Pi16(8)	0	1	2
Adcy6(6)	0	0	1	Gm26518(3)	0	2	1	Pisd(3)	0	1	1
Ajm1(10)	0	4 B	2	Gm26576(5)	0	3	1	Plekhh3(7)	0	1	1
Ankrd24(7)	0	1	2	Gm28511(7)	0	2	2	Plxna4(7)	0	1	1
Apoe(5)	0	1	1	Gm29050(6)	0	1	0	Pou2f2(5)	0	0	1
Arhgap8(7)	0	1	1	Gm36823(3)	0	1	0	Prima1(5)	0	1	2
Arhgap9(11)	0	1	0	Gm38708(5)	0	1	0	Proser1(9)	1	5	1
Atpif1(2)	0	1	0	Gm4278(3)	1	1	1	Prpf4b(4)	0	1	0
B230208B08Rik(3)	0	1	1	Gm43401(5)	4 CR	5 F	3	Qprt(4)	4 CR	4 M	2
Bik(9)	1	4	2	Gm44448(3)	0	1	2	Ralgps1(5)	0	2	1
Bola1(5)	1	1	0	Gm48693(18)	0	1	1	Rapgefl1(9)	0	1	2
Borcs8(7)	0	1	0	Gm49012(3)	0	1	1	Rasef(16)	0	2	3
Brinp2(5)	1	2	2	Gm49329(10)	0	3	3	Rbpms2(8)	3 CR	5	0
C1qtnf1(5)	1	2	1	Gm49383(9)	0	1	1	Rcan1(5)	0	1	1
Cadm1(21)	0	4	4	Gm6226(4)	0	1	1	Rfx2(8)	0	3	3
Calb2(18)	0	4 M L	6 T	Gp5(6)	0	1	0	Rgma(2)	0	1	2
Carlr(7)	0	1	1	Grk6(2)	0	2	1	Rhot1(13)	0	2	1
Ccdc146(6)	0	4	2	H2‐Bl(14)	0	4	4	Rimbp3(12)	1	2	2
Ccdc30(4)	0	1	2	Hdgfl2(8)	3 CR	5	0	Rph3al	0	1	0
Ccr7(3)	1	1	0	Hsf4(17)	0	4	3	Rps6ka4(13)	0	3	2
Cd5(5)	0	3	3	Hspb6(8)	0	1	1	Rusc2(5)	0	2	2
Cdh11(6)	0	1	0	Iffo2(7)	0	7 M L G	7 M L G	Scamp2(3)	2	2	0
Cdh8(24)	0	7	7	Igfbp6(5)	0	1	2	Scara5(3)	1	3	1
Ciita	0	1	0	Il17rd(7)	0	1	1	Sirt4(9)	0	2	1
Cldn4(11)	1	4	3	Itgb4(7)	0	6 G	5 G	Slc16a12(5)	2	3	0
Clvs2(12)	3 CR	5	5 B	Kank2(5)	0	1	1	Smim10l1(17)	0	2	3
D130043K22Rik(2)	0	1	1	Kcnn2(2)	0	0	1	Sox30(12)	1	4	2
Dapk1(5)	0	1	0	Kcns1(32)	2	9 CR In	8	Spats2(6)	0	1	0
Ddr1(5)	0	1	0	Kctd15(3)	0	1	1	Srcin1(11)	0	1	2
Dgkg(9)	0	3	3	Khdrbs2(11)	0	1	4	Stard3(3)	0	3	0
Dido1(3)	0	2	0	Kiz(4)	0	0	1	Tiam1(4)	0	1	1
Dnajb2(4)	4 CR	4	3	Klf14(10)	1	1	0	Tlx3(7)	0	2	2
E2f4(7)	0	2	3	Lncpint(8)	0	3	3	Tmem121b(10)	0	2	3
Egr3(10)	0	4 In	3	Lncppara(15)	3 CR	5	2	Tnfaip2(7)	0	2	3
Epop(3)	0	2	0	Lrba(12)	0	1	2	Tnip1(3)	0	3 In	3 In
Evx2(18)	1	5	3	Lrrc75a(6)	0	1	2	Tns1(8)	0	3	1
Exoc3l2(2)	0	1	1	Lsr(10)	0	1	1	Ttc39b(12)	0	3	4
Fam169a(7)	0	2	3	Lynx1(4)	0	1	1	Wasf3(8)	6 CR	5 CR	0
Fam84b(5)	3 CR	3	2	Map10(34)	3 CR	4	1	Wnk4(2)	0	2	1
Fbxo40(6)	0	2	1	Mbd2(8)	0	5 In	7 In	Wnt3a(9)	0	3 D	1
Fgf8(7)	0	1	2	Mgat5b(3)	0	0	1	Wscd1(6)	0	1	1
Fosb(6)	0	2	0	Mn1(5)	2	4 M D G	2	Yars2(9)	1	1	1
Foxl2os(17)	1	3	2	Morn3(6)	0	2	1	Zfhx3(10)	0	1	1
Fzd2(10)	0	2	5	Mpp5(7)	0	0	2	Zfp148(4)	0	1	1
Gabra5(14)	0	2	3	Mtcl1(2)	1	2	1	Zfp64(9)	0	1	1
Gas7(6)	0	1	1	Mymx(11)	0	1	2	Zic1(12)	5 CR	3 CR	1
Gdf3(6)	0	3	1	Nbea(13)	1	3	1	Zmiz1os1(2)	1	2	1
Gga1(6)	0	2	0	Nfic(16)	3 CR	7 B	2	Zscan2(16)	0	5	5
Gkn3(6)	0	1	1	Nhlrc2(11)	0	3	2	Zswim6(5)	0	2	2

*Note:* The numbers in the columns are the number of significant CpG sites within each gene. The table lists variables that are significant at 3 or more significant CpG sites within a gene. Variables are denoted as CR—calorie restriction, M—ln(mass), L—ln(leptin), In—ln(insulin), Ig—ln(IGF), B—ln(BMR), P—ln(PA), T—ln(Tb), F –FAA, Ca—catalase, S—SOD, D—DNA damage, Fg—fasting glucose and G—area under the curve of glucose tolerance test (GTT).

**FIGURE 2:**
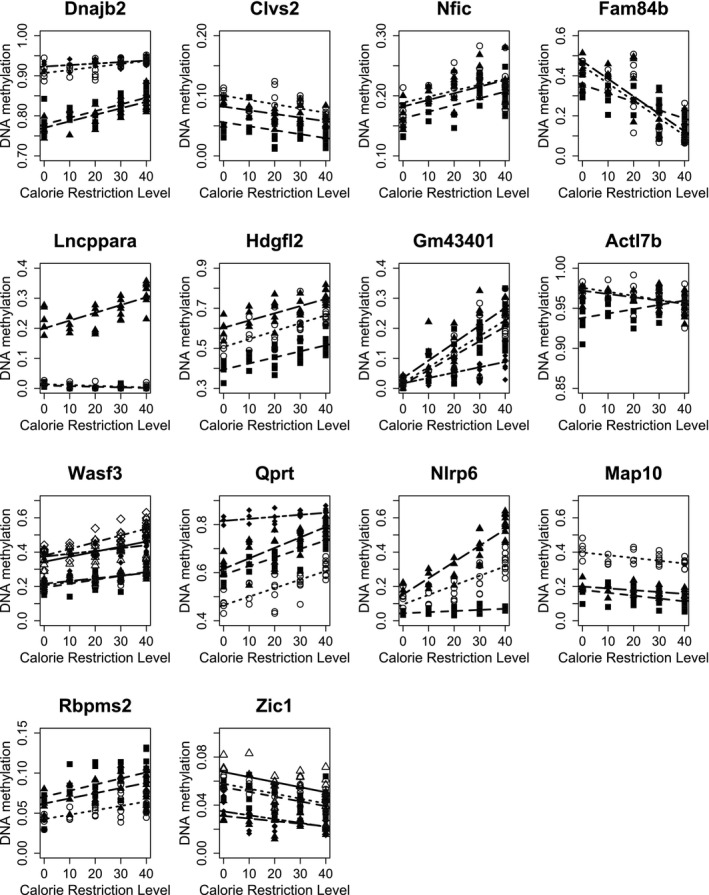
DNA methylation variation with calorie restriction (CR) level for mice aged 24 m following 19 m CR, for 14 genes containing three or more significant CpG sites. Calorie restriction level 0 is 12‐h ad‐libitum feeding. The number of significant CpG sites varied between 3 and 6. The different symbols represent (Calorie Restriction level, DNAm) measurements at a given significant CpG site within the gene, and a best‐fit line is drawn for each CpG site.

Univariate linear regression of DNAm against CR level revealed 80 significant intragenic CpG sites (Table 1). Some genes contained multiple significant CpG sites, which may be indicative of a greater DNAm heritability and role in gene expression (Lövkvist et al. 2016). This leads us for practical visualisation purposes to focus on genes with three or more significant CpG sites. For the fourteen genes (Figure 2) with three or more significant CpG sites, the gradient of the regression of twelve of them was all positive (eight) or all negative (four). Two genes, each with 3 significant CpG sites, have positive and negative regression gradients; for both of these genes, the two CpG sites located closest to each other had the same sign regression gradient.

Multiple linear regression of DNAm against all possible combinations of predictor variables revealed 370 significant intragenic CpG sites. The most frequently occurring significant predictor variables across these CpG sites were ln(insulin) and CR (12% each of all significant predictor variables), ln(leptin) and ln(mass) (10%), ln(BMR) and catalase and DNA damage (9%). No single predictor variable dominated, and although ln(leptin) and ln(mass) were strongly correlated with CR, the number of occurrences of ln(insulin) and catalase may indicate other factors beyond CR control of DNAm at some CpG sites.

**FIGURE 3:**
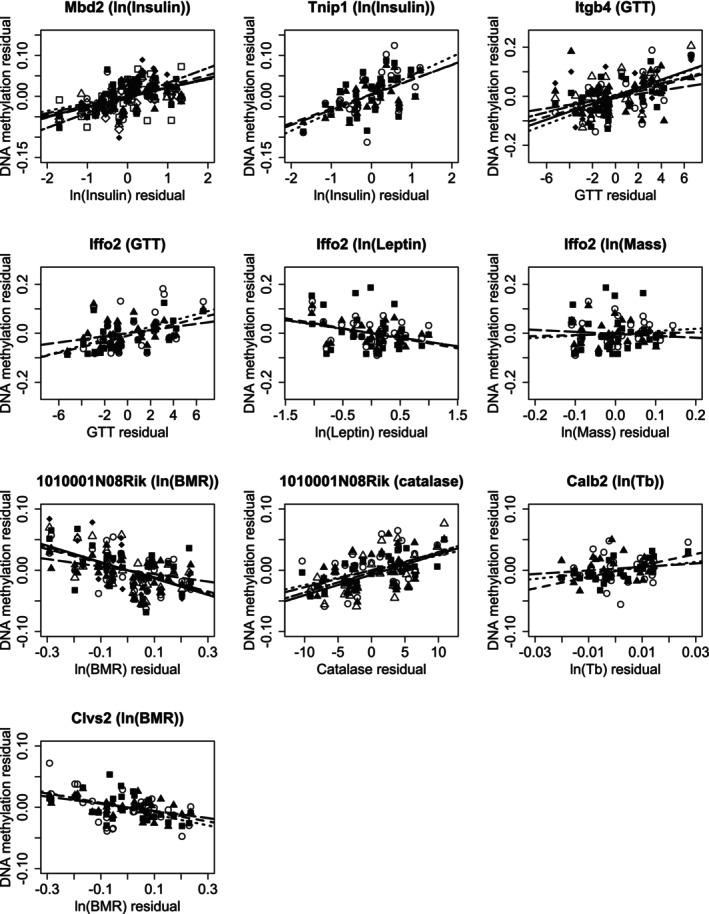
DNA methylation variation residual values after accounting for CR for mice aged 24 m following 19 m CR for genes containing three or more significant CpG sites for which the same predictor variable residual to CR is significant. The charts show the three most significant CpG sites for each gene/predictor variable combination. The different symbols represent a set of (predictor variable residual, DNAm residual) at each CpG site and together with a best‐fit line through each set of data.

Multiple linear regression of DNAm residual values against predictor variable residual values after removing the shared effect of CR revealed 280 significant intragenic CpG sites. Seven genes (Figure 3) contained three or more significant CpG sites for which the same predictor variable was significant. Two genes had three or more significant occurrences of ln(insulin), two for GTT, two for BMR, and one each for ln(mass), ln(leptin), Tb, and catalase. Gene 1010001N08Rik had more than 3 significant occurrences of BMR and catalase. Iffo2 had three or more occurrences of ln(mass), ln(leptin), and GTT. The other five genes had three or more significant occurrences of only one predictor variable.

Results were also obtained for intergenic CpG sites. Of the 2045 CpG sites at which DNAm was measured, 24% were in intergenic regions. Only 9% of the CpGs that were significant in univariate linear regression were intergenic. For intergenic regions, 23% of significant CpGs from multiple linear regression were also observed, and 23% for linear regression of residuals. There was no significant difference between the relative occurrence of different predictor variables in the intra‐ and intergenic regions for multiple linear regression of absolute data (χ^2^_13_ = 12.95, *p* = 0.452) or residuals (χ^2^_12_ = 16.88, *p* = 0.154). When viewed at the combined intra‐ and intergenic level, no individual predictor variable dominated; the most frequent occurrences were catalase, ln(insulin), ln(mass), DNA damage, ln(BMR), and ln(leptin), each accounting for between 9% and 13% of all predictor terms.

**FIGURE 4:**
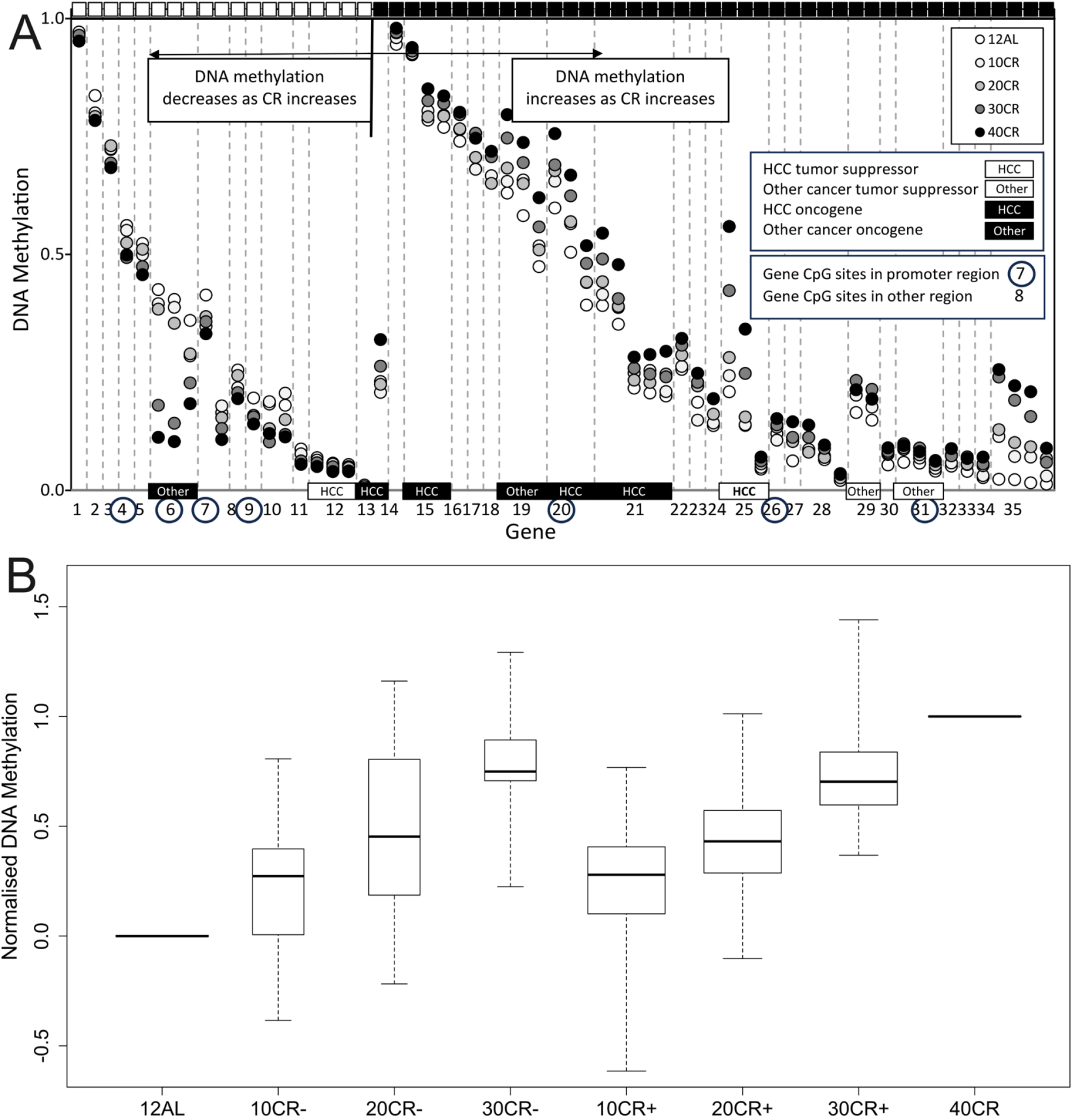
DNA methylation over graded calorie restriction (CR) levels of 24‐month‐old male C57BL/6J mice following 19 months CR. (A) DNA methylation by individual CpG site. Symbols: White 12AL, light grey 10CR, mid grey 20CR, dark grey 30CR, black 40CR. Black/white squares indicate DNA methylation increasing/decreasing from 12AL to 40CR. CpG sites with outlier DNA methylation values and CpG sites with significantly non‐normal data Shapiro–Wilk test with Benjamini‐Hochberg correction for multiple tests were excluded. The CpG sites illustrated are those where there was a significant difference between DNA methylation for 12AL and 40CR levels. The gene/intergenic regions are: 1 Actl7b, 2 Cldn4, 3 Mtcl1, 4 C1qtnf1, 5 intergenetic4, 6 Fam84b, 7 Map10, 8 Sox30, 9 Klf14, 10 Kcns1, 11 Foxl2os, 12 Zic1, 13 Lncppara (one increasing and one decreasing CpG site), 14 Gm4278, 15 Dnajb2, 16 intergenetic1, 17 Mn1, 18 Scamp2, 19 Qprt, 20 Hdgfl2, 21 Wasf3, 22 Nt5c1a, 23 Bik, 24 Ccr7, 25 Nlrp6 (conflicting literature regarding role in HCC), 26 Nbea, 27 Pde4dip, 28 intergenetic5, 29 Nfic, 30 Zmiz1os1, 31 Rbpms2, 32 Adamtsl5, 33 Slc16a12, 34 intergenetic3, 35 Gm43401. All significant CpG within a gene are plotted consecutively. Along the bottom axis we identify genes known to be tumour suppressors or oncogenes for hepatocellular carcinoma (HCC) and for other cancers (Other). A circled number indicates the CpG sites for the gene are in the promoter region. (B) The average DNA methylation for each CR level normalised between the average of the 12‐h ad‐lib (12AL) group (a normalised value of zero) and the average of the 40% calorie restriction (40CR) group (normalised value of one). Axis label CR levels with a—sign indicate DNA methylation decreases from AL12 to 40CR and a + sign indicates DNA methylation increases. The boxes are interquartile range (IQR) and whiskers extend to the data extremes.

Linear regression located CpG sites that exhibit a change in DNAm across the range of CR levels. By using a limited number of CR levels, it is possible to determine whether the rate of change in DNAm is linked to a particular level of CR, or whether progressive CR results in gradual change in DNAm. CpG sites may have highly different values of DNAm and may show different directionality as CR increases (Figure 4A). To assess graded change in DNAm with CR DNAm values were normalised between 0 representing the DNAm for 12AL and 1 representing the DNAm for 40CR. The normalised DNAm values at each level of CR are shown in Figure 4B. The 10CR, 20CR, and 30CR data are shown separately for CpG sites where DNAm increased or decreased with CR. Linear regression of both increasing DNAm with CR, *F*_1,127_ = 78.8, *p* < 0.0001, and decreasing DNAm with CR, *F*_1,55_ = 29.31, *p* < 0.0001, showed significant relationships. In addition, there was no difference between the mean normalised values of DNAm whether increasing or decreasing with CR (10CR: *t*(60) = 0.19, *p* = 0.85; 20CR: *t*(60) = −0.99, *p* = 0.33; 30CR: *t*(60) = −0.11, *p* = 0.92). For our data, it is reasonable to view all normalised DNAm values as a single dataset irrespective of whether a CpG site has increasing or decreasing DNAm with CR.

### 2.2 Epigenetic Ageing

**FIGURE 5:**
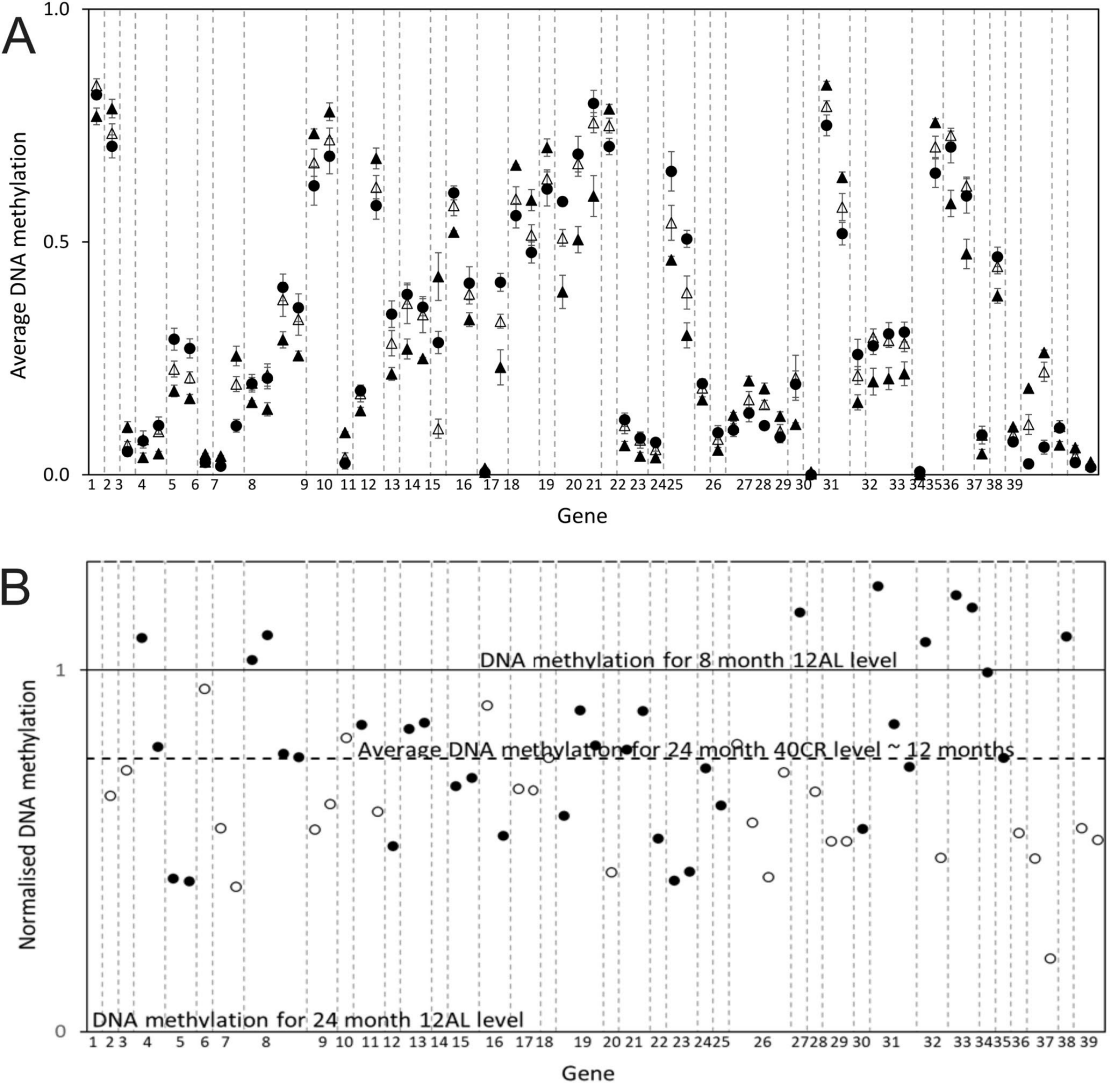
Slowing of Epigenetic Ageing. (A) values for 12‐h ab‐lib (12AL) feeding and 40% calorie restriction (40CR) at 24 months after 19 months calorie restriction (CR) relative to 12AL feeding at 8 months after 3 months CR (filled triangle 24‐month 12AL, open triangle 24‐month 40CR, filled circle 8‐month 12AL, error are SEM). (B) Normalised DNA difference of 24‐month 40CR samples versus 8‐ and 24‐month 12AL (filled black circle DNA methylation decreases from 8‐month to 24 month ad‐lib feeding, open black circle DNA methylation increases from 8 month to 24 month ad‐lib feeding). One data point of 2.31 (decreasing) for gene Fam84b is omitted from the graph. The average position of all 24‐month mice 40CR data is equivalent to mice aged about 12 months. The gene/intergenic regions are: 1 Dnajb2, 2 Brinp2, 3 Clvs2, 4 Adamtsl5, 5 Nfic, 6 Tlx3, 7 Sox30, 8 intergenic1, 9 Stard3, 10 Fzd2, 11 intergenic2, 12 Grk6, 13 Scara5, 14 Fam84b, 15 Lynx1, 16 Lncppara, 17 Gm26518, 18 Ddr1, 19 Hdgfl2, 20 Cd5, 21 Slc16a12, 22 Dido1, 23 Proser1, 24 Nbea, 25 intergenic3, 26 intergenic4, 27 intergenic5, 28 Gm10463, 29 Cldn4, 30 intergenic5, 31 Wasf3, 32 intergenic6, 33 Qprt, 34 Nlrp6, 35 intergenic7, 36 Cdh8, 37 Map10, 38 Rbpms2, 39 Zic1.

Epigenetic ageing in response to CR was evaluated by whether the change in DNAm with increasing CR was in the same direction as the change in DNAm between the 24‐month 12AL and the 8‐month 12AL group. At 65 CpG sites there was a significant difference in DNAm between 8‐month and 24‐month 12AL and between 24‐month 12AL and 40CR (Figure 5A). The same data are shown in normalised form in Figure 5B where the difference between 24‐month 12AL and 40CR is plotted relative to the difference between 24‐month 12AL level (normalised value 0) and 8‐month 12AL level (normalised value 1). In all cases the DNAm value for 24‐month 40CR moved away from 24‐month 12AL towards, and in some cases beyond, 8‐month 12AL. This occurred whether the difference in DNAm between 24‐month and 8‐month 12AL levels was increasing or decreasing. The average movement of the 24‐month 40CR samples away from 24‐month 12AL was 0.75 of the DNAm difference between 24‐month and 8‐month 12AL levels. If interpreted as linear change the CpG sites for 24‐month 40CR had an average epigenetic age of 12‐months 12AL. Although the requirement for both *t*‐tests to be significant limited the number of CpG sites to 65, 310 sites were significant for at least one of the *t*‐tests. For these 310 CpG sites, 284 had directional change consistent with slowing of epigenetic ageing for 24‐month 40CR relative to 24‐month.

### 2.3 Collinearity in Multiple Linear Regression

**FIGURE 6:**
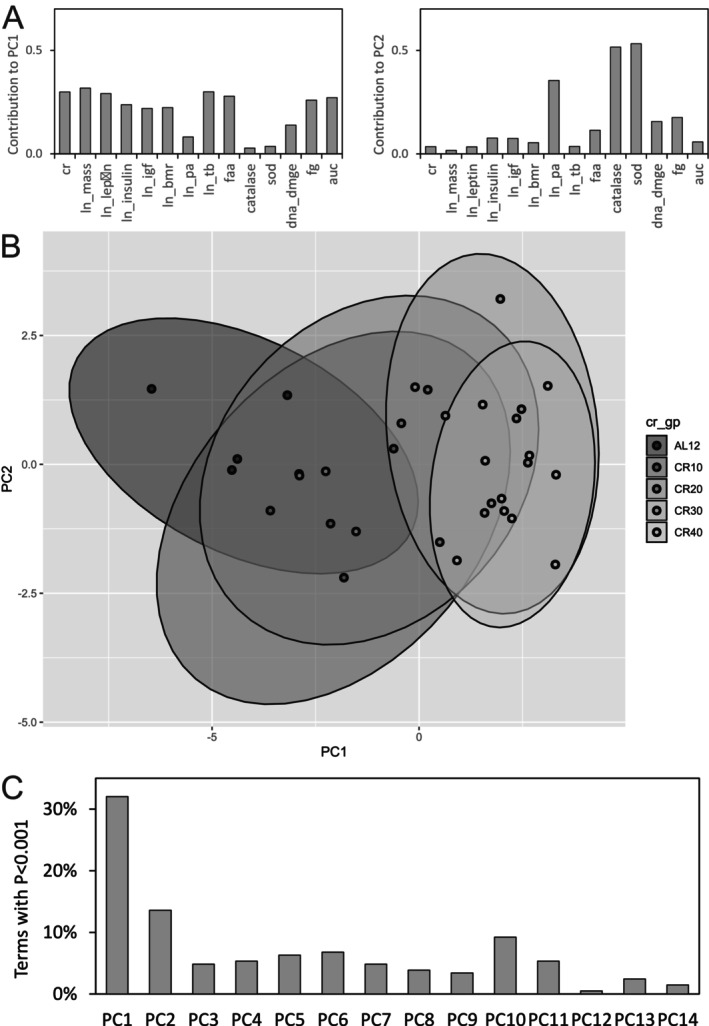
PCA analysis. (A) Relative contribution of each measured predictor variable to principal components 1 and 2. (B) Predictor variables recast to PC1 and PC2. The ellipses are 95% confidence areas. (C) Multiple linear regression for each of 2045 CpG sites using principal components as predictor variables showing the relative number of occurrences of each PC with *t*‐test *p* < 0.001.

To further investigate the issue of collinearity among predictor variables in multiple linear regression, a principal components analysis (PCA) was carried out. Figure 6A shows the contribution of each predictor variable in PC1 and PC2. All predictor variables except catalase, superoxide dismutase, and physical activity contributed similar weight to the first principal component (PC1). Catalase, superoxide dismutase, and physical activity provided the greatest weighting for the second component (PC2). Together PC1 and PC2 accounted for 59% of the variation in DNAm. Figure 6B shows that individuals at different levels of CR clustered together at different locations in the space defined by PC1 and PC2. The initial movement in PC1‐PC2 space away from 12AL feeding to 10CR and 20CR is widely scattered, with more substantial separation, at 95% confidence, seen between 12AL feeding and 30CR and 40CR groups. Multiple linear regression was rerun using PC components; Figure 6C, shows PC1 as the most dominant term in the lowest AIC (optimum) models.

### 2.4 DNAge

DNAge (biological age based on DNAm) was estimated from a proprietary DNAm‐based algorithm. One mouse in the 30CR group was determined from necropsy examination to have liver neoplasia and also to account for 42% of all DNAm outliers relative to a normal distribution of residual values. The DNAge of this mouse was 131 weeks which is a significant outlier (two‐sided Grubbs test *G* = 2.44, *G*_crit_ = 2.03, α = 0.05, *p* < 0.0001). Linear regression after removing the outlier indicated a significant relationship between DNAge and CR level, *F*_1,30_ = 20.71, *p* < 0.0001 (Supporting Information Figure S1).

## 3 Discussion

CR lengthens lifespan in a diverse range of species (Green et al. 2022), with the lifespan response positively related to the level of CR (Di Francesco et al. 2024; Mitchell et al. 2023; Speakman et al. 2016). Epigenetic responses occur over the lifespan, and CR has been shown previously to protect against age‐related DNA methylation drift (Maegawa et al. 2017). Using graded levels of CR over 19 months in male C57BL/6J mice, a progressive change in DNA methylation in the liver was observed. For those CpG sites where significant DNAm changes occurred, both the individual responses (Figure 4A) and averaged normalised responses (Figure 4B) were positively associated with CR level.

### 3.1 CpG Sites With Significant DNAm Variation

Although DNAm changes at a single CpG site have been linked to disease, numerous studies have shown that DNAm alteration at multiple CpG sites in a gene may be functionally more important than alterations at a single site (Lövkvist et al. 2016; Seale et al. 2022). In view of our general exploration across many CpG sites for practical purposes, we focused our visualisation on genes where three or more CpG sites showed significant changes in DNAm. We found fourteen genes that had three or more CpG sites with significant variation in DNAm with CR level (Figure 2) and seven genes where DNAm varied significantly with other predictor variables (Figure 3). Clvs2 was significant for both CR and ln(BMR), so we include 20 genes in our figures. A literature search of these 20 genes found 9 that promote or suppress hepatocellular carcinoma (HCC): Dnajb2, Hdgfl2 (synonym Hrp2), Lncppara, Nlrp6, Wasf3 (Wave3), Zic1, Calb2, Itgb4, Mbd2; three with an active role in other cancers: Fam84b (Lratd2), Qprt, 1010001N08Rik (LncGata6); five with a role in other liver disease, cell protection or cell proliferation: Clvs2, Tnip1, Nfic, Rbpms2, Iffo2; and three with no information specific to liver function: Map10, Actl7b and Gm43401 (Table 2). Supporting Information Table S3 summarises the literature search of Supporting Information Table S4, which provides links between these genes and other cancers.

**TABLE 2:** Hepatic function of genes where linear regression found three or more CpG sites for which DNA methylation is significantly dependent on a single predictor variable.

Gene (synonym)	Predictor variable	Function(s)	Liver disease (Hepatocellular carcinoma (HCC), Gastric Cancer (GC))
Actl7b	CR	Expressed predominantly in the testis, exact function is not known.	No information on hepatocytes but known to regulate spermiogenesis and required for male fertility
Dnajb2	CR	Contributes to proteasomal degradation of misfolded proteins	High expression is unfavourable in HCC
Fam84b (Lratd2)	CR	Located in cytoplasm and plasma membrane	Collaborates with MyC and POU5F1B in cancer proliferation but no direct evidence for HCC
Gm43401	CR	No information	No information
Hdgfl2 (Hrp2)	CR	Promotes the repair of DNA double‐strand breaks. Cellular growth control.	Promotes cell growth in HCC
Lncppara (Mirlet7bhg)	CR	Predicted to be involved in post‐transcriptional gene silencing	Facilitates HCC
Map10	CR	Regulation of cell division and promotes microtubule stability	No information on hepatocytes
Nfic	CR	Cellular transcription factors and replication factors for adenovirus DNA	Regulate hepatocyte proliferation during liver regeneration and an antioncogene for lung squamous cell carcinoma
Nlrp6	CR	Mediates inflammasome activation, recognises & binds specific pathogens	HCC oncogene or tumour suppressor? Higher immune cell infiltration vs. inhibiting macrophage infiltration and suppressing phagocytosis
Qprt	CR	Involved in catabolism of quinolinate, a potent exitotoxin to neurons	Enhances cancer invasiveness for breast cancer and similar mechanisms observed in HCC
Rbpms2	CR	Regulates smooth muscle cell differentiation and proliferation	Overexpressed in liver cells resistant to Hepatitis C antiviral drug Ribavirin. In other tissue, downregulation promotes GC
Wasf3 (Wave3)	CR	Plays a role in the regulation of cell morphology and cytoskeletal organisation	A master regulator for metastatic signalling. Promotes HCC invasiveness and metastasis
Zic1	CR	Developmental protein involved in neurogenesis and DNA‐binding	HCC tumour suppressor, hypermethylation is correlated with tumour size
Clvs2	CR, BMR	Require for normal morphology of late endosomes and lysosomes in neurons	Upregulated in *Clonorchis sinensis* infected, and lenvatinib resistant HCC
1010001N08Rik (LncGata6)	BMR, Catalase	Expressed in the embryonic heart and during cardiomyocyte differentiation	Activates Ets homologous factor (EHF), and EHF knockdown decreases liver metastasis of colorectal carcinoma cells.
Calb2	Tb	Intracellular calcium‐binding protein, role in message targeting	Activates signalling pathway to promote HCC metastasis
Iffo2	GTT, Mass Leptin	Role in molecular structure of cells	Differentially expressed in significant liver fibrosis
Itgb4	GTT	Activates ERBB receptors and induction of acetylcholine receptor expression	Mediates the pathway to promote HCC metastasis. ERBB receptors are linked to human cancer pathogenesis
Mbd2	Insulin	Binds CpG islands in promoters where the DNA is methylated	Regulates transcription onset of HCC critical genes and induces invasiveness
Tnip1	Insulin	Inhibits NF‐κB activation and TNF‐induced NF‐κB dependent expression	Protects hepatic cell injury through inhibition of pro‐inflammatory cytokines

*Note:* Gene function from www.proteinatlas.org and www.genecards.org. Liver disease references in main text.

Abbreviations: GC, gastric cancer; HCC, hepatocellular carcinoma.

The CpG sites for genes that promote or suppress HCC or other cancers where we find a significant difference between DNAm for 12AL and 40CR groups are shown in Figure 4A. The HCC tumour suppression gene (Zic1) DNAm decreases as CR increases and for all four HCC oncogenes (Lncppara, Dnajb2, Hdgfl2 and Wasf3) the opposite trend was observed. Assuming methylation reduces gene expression, then all five genes associated with HCC indicate a reduction in HCC risk as CR increases. These four HCC oncogenes make up four of the top five genes with the largest changes in DNAm between 12AL and 40CR among the 23 genes we identified with DNAm increasing as CR increases. For oncogene and tumour suppression genes for cancer in other tissues we observed a mixed response of DNAm increasing or decreasing with CR. In general for both HCC and other cancers we found that tumour suppression genes have lower levels of DNAm, less than 0.2, whereas oncogene DNAm levels vary between about 0.2 and 0.9 and with greater change between 12AL and 40CR groups. We did not find DNAm variation for CpG sites in promoter regions to be significantly different from other CpG sites. This may be because all 2045 CpG sites in our analysis are known to show DNAm variation with age. In addition, our analysis further refined the CpG sites under consideration to those where we observed significant change between 12AL and 40CR groups. In this context it is difficult to draw conclusions about relative DNAm changes for CpG sites in promoter regions and those in other regions.

After accounting for the effects of CR on DNAm and predictor variables, multiple linear regression revealed significant relationships between DNAm and predictor variables. The predictor variables can be considered in three groups: those with insulin and glucose intolerance (Mbd2, Tnip1, Itgb4, and Iffo2), those with BMR (1010001N08RiK and Clvs2), and those associated with Tb (Calb2). Mbd2 and Tnip1 have significant relationships between DNAm variation and insulin type 2 diabetes. Similarly Itgb4 and Iffo2 DNAm correlate with glucose intolerance with Itgb4 upregulated in diabetic glomeruli and Iffo2 methylation associated with dietary glycaemic load. The associations with insulin make Mbd2 and Tnip1 potentially significant players in the nutrient sensing insulin/insulin‐like growth factor‐1 signalling pathway (IIS). Attenuated IIS activity is an evolutionarily conserved pathway thought to mediate anti‐ageing effects across metazoans (Fontana et al. 2010; Hwangbo et al. 2020), with CR strongly linked to improved IIS (Mitchell, Delville, Konstantopedos, Hurst, et al. 2015). BMR response to CR is complex. Tissues respond in different ways as the individual needs to regain its energy balance under reduced energy input. Liver mass is a major predictor of BMR under CR (Mitchell et al. 2017). The long non‐coding RNA gene 1010001N08Rik was significant for DNAm variation with BMR and catalase. Functionally, 1010001N08Rik enhances activation of the highly conserved Wnt signalling pathway which, among many other impacts, affects the liver by modulating nutrient uptake by regulating macropinocytosis (Colozza and Koo 2021). For 1010001N08Rik, catalase was also significant for DNAm variation, usually in linear combination with BMR. Both BMR and catalase decrease with increasing levels of CR, but their residuals provide contrasting responses: BMR increases and catalase decreases. Catalase in Zebrafish liver is regulated by Wnt family protein Wnt10b (Li et al. 2022). It appears that Wnt signalling may provide a common thread between BMR and catalase responses. Alongside 1010001N08Rik, Clvs2 was also significant for DNAm variation with BMR residuals. Clvs2 functions in a pathway between early endosomes and mature lysosomes which are integral to macropinocytosis regulated by Wnt. Overall, the genes which show DNAm variation with BMR residuals have associations with the Wnt signalling pathways, which itself adapts dynamically to nutrient availability.

Calb2 is associated with inflammatory responses and DNAm residuals were significantly correlated with Tb residuals at 3 CpG sites. At each CpG site at which Tb is a significant predictor variable, it is combined with several other predictor variables in the optimum model, with the average contribution of Tb being about 36%. The relatively low contribution of Tb to overall model prediction and the spread between CpG site gradients (Figure 3) increase the uncertainty in assigning a functional linkage between Calb2 DNAm and Tb.

### 3.2 Slowing of Epigenetic Ageing

For a subset of the sampled CpG sites we found a significant difference in the DNAm values between 8‐month 12AL and 24‐month 12AL. Many of these significant CpG sites were also significant for the graded CR DNAm response. The nine genes with significant variation of DNAm with CR implicated in HCC promotion or suppression (Table 2) also have CpG sites that are significant for slowing of epigenetic ageing. When comparing DNAm values from 24‐month 12AL mice to 24‐month 40CR the change was, without exception, in the same direction as the DNAm change from 24‐month 12AL mice to 8‐month 12AL (Figure 5), that is, slowing of epigenetic ageing. Our results showed an average reduction in epigenetic age from 24 months for 12AL to 12 months for 40CR similar to a previous study based on only 4 CR and 4 AL mice, which showed a reduction from 22 months for controls to 12.6 months when food was reduced to 60% of their aged‐matched controls (Wang et al. 2017).

### 3.3 Collinearity Among Predictor Variables

Many changes are brought about by CR in the morphological, physiological and behavioural activities of mice, and as we have shown here, the DNAm landscape also changes. During multiple linear regression of measured values of DNAm and predictor variables, it is likely that the response to some factors may be obscured by the non‐orthogonality of the predictor variables. We overcome collinearity effects in the first instance by multiple linear regression on residuals, which removes the effect of CR from both predictor and outcome variables, and secondly by PCA analysis. We found that the first principal component (PC1) accounted for 50% of total variation, and the greatest contributions to PC1 were from CR, body mass, body temperature, three circulating hormones: leptin, insulin and insulin like growth factor‐1, two factors that relate to glucose intolerance: fasting glucose and area under curve, together with basal metabolic rate and food anticipatory activity (Figure 6A). The importance of these factors to explaining DNAm variation mirrors their suggested importance in beneficial effects of CR as observed in C57BL/6J mice under 3 months graded CR (Derous et al. 2016, 2017; Mitchell, Delville, Konstantopedos, Derous, et al. 2015; Mitchell, Delville, Konstantopedos, Hurst, et al. 2015; Mitchell, Tang, Kerbois, Delville, et al. 2015; Mitchell et al. 2017; Mitchell, Delville, et al. 2016).

### 3.4 Outliers

Many DNAm outliers define epigenetic defects and are selected for during cancer progression and may predate the emergence of tumours (Baylin et al. 2001; Teschendorff et al. 2016). We found that DNAm outlier values tended to cluster in particular genes associated with disease observations from necropsy results or to an extreme value among predictor variables. For example, a mouse with liver neoplasia identified from necropsy examination accounted for 42% of all DNAm outliers; this individual had 19 genes (many linked to cancer) where every CpG site was an outlier whereas it also had 82 genes with zero outliers. Another mouse, with lung neoplasia, had 50% of its outliers within two genes, Lrba and Tnfaip2, both of which are linked to lung cancer (Li et al. 2020; Wang et al. 2004). Outliers for predictor variables may also be indicative of disease. The mouse with liver neoplasia above had an outlier value for the predictor variable DNA damage. Another mouse was an outlier for extreme high mass although with no disease identified by necropsy examination but we found all CpG sites in the Apoe gene were DNAm outliers and this gene is linked to hyperlipoproteinemia and in turn associated with obesity.

Our focus was to ascertain the effect of CR on DNAm rather than as a diagnostic tool for malignant tissue. We recognise the diagnostic value of DNAm outliers but also that neoplasia appears to be an additional variable that obscures the underlying patterns due to CR. We identified DNAm outliers by statistical test and removed them. In our univariate analysis 2.3% of a total of 67,485 DNAm observations were outliers, with 79% of these from just 5 individuals. Outliers removed from DNAm data for the remaining 28 mice account for less than 0.5% of the total. Similarly, outliers among predictor variables were removed by the same methodology. Six data points were removed from a total of 421 measurements (1.4%). The removal of predictor variables impacts on multiple linear regression on measured data and residual analyses but not univariate CR regression.

DNAm outliers for the mouse with liver neoplasia, an age‐related condition, were more frequent on genes that contained at least one significant CpG site for DNAm variation with CR than for genes which did not contain a significant CpG site (χ^2^_1_ = 26.69, *p* < 0.0001). The outliers on significant genes in Figure 2 were all located, relative to the regression line, in a position consistent with an extremely aged individual based on our observations of slowing of epigenetic ageing with CR; additionally, the 4 CpG sites for Wasf3 and the 5 sites for Zic1 show the outliers to be hypo‐ and hypermethylated respectively, consistent with published HCC biomarkers (Supporting Information Table S3, Supporting Information Figure S2). This reinforces our view that the presence of neoplasia is an additional variable that strongly influences DNAm pattern and that removing outlier data is appropriate.

### 3.5 Epigenetic Clocks

Moving to consideration of multiple CpG sites as components of epigenetic clocks, there are several examples where clocks have provided high accuracy in estimating chronological human and mouse age by sampling methylomes (Horvath and Raj 2018; Stubbs et al. 2017; Wang et al. 2017). However, in mice and humans, a clock that is highly accurate for estimating age might not be most efficient for detecting the beneficial effects of anti‐ageing interventions, perhaps due to the choice of regression method or the population of sampled CpG sites (Thompson et al. 2018). The extent and duration of CR relative to baseline may also be important; in humans, epigenetic clocks could not detect a change in biological age over a two‐year CR trial (Belsky et al. 2022; Waziry et al. 2023), but a PCA approach successfully detected beneficial effects of CR based on the same data (Fong et al. 2024). Here, the DNAge clock showed slowing of biological ageing due to CR in mice, but at smaller magnitude than the initial change between chronological and biological age for 12AL. It may be that the 12‐h fasting period inherent in the 12AL regime confers health benefits and slowing of epigenetic ageing, as has been reported previously (Pak et al. 2021).

In summary, in male C57BL/6J mice, CR produced a slowing of ageing based on age‐associated changes in DNAm. The DNAm changes increased progressively throughout the CR range from 12AL to CR40. The strongest DNAm responses, if based on the number of CpG sites with significant DNAm alteration, were dominated by genes with an established association with hepatocellular carcinoma promotion or suppression. Other genes with multiple significant CpG sites were associated with other liver diseases such as hepatitis C, lung squamous cell carcinoma, and liver fibrosis. For our data, we observed that genes with strong DNAm responses to CR also appear more likely to exhibit outlier DNAm points from individuals for which neoplasia is present. We conclude that removal of outliers is appropriate to reveal the underlying DNAm variation with CR. For a subset of genes, a significant DNAm response occurred with changes in the area under the curve of glucose tolerance tests and with circulating insulin levels consistent with the insulin/IGF signalling (IIS) nutrient sensing pathway impact on ageing.

## 4 Methods

### 4.1 Animals

For full details of experimental design refer to (Mitchell, Tang, Kerbois, Delville, et al. 2015; Mitchell et al. 2023). Procedures were carried out under Home Office Licence (PPL60/366) which was reviewed and accepted by the University of Aberdeen's Welfare and Ethical Review Body. Studies were compliant with the Animals (Scientific Procedures) Act 1986 and carried out in accordance with the ARRIVE Guidelines (Percie du Sert et al. 2020).

Male C57BL/6J mice (Charles River, Ormiston, UK) were acclimated to single housing for 2 weeks with free access to food and water. Prior to the study, mice were implanted intraperitoneally with transmitters recording minute by minute physical activity and body temperature (VitalView telemetry and data acquisition system, MiniMitter, OR, USA) (Mitchell, Delville, Konstantopedos, Derous, et al. 2015).

### 4.2 Diet

Animals were fed D12450B for the 8‐month CR study and D12450H for the 24‐month CR study (Research Diets, USA). While the macronutrient content of both diets was 70% carbohydrate, 20% protein and 10% fat (kcal%), D12450H contained reduced sucrose (17% versus 35% in D12450B). During the CR phase of the 24‐month study, the 30CR and 40CR levels were fed D13020504, which matched D12450H with a 40% increase in the vitamins mix to avoid malnutrition in mice fed higher levels of CR; this data was previously published (Mitchell et al. 2023).

Over a 2‐week baseline period, all mice were fed 12‐h ad libitum with food provided only in the dark phase. This is in accord with the nocturnal feeding of mice and ensures all mice are similarly fasted at time of kill (Mitchell, Tang, Kerbois, Delville, et al. 2015). Food provided to the CR groups was calculated over the baseline period from individual average daily 12AL food intake. All mice were fed directly before the start of the dark phase with food removed from 12AL mice at the start of the light phase. Body mass was measured daily immediately prior to feeding.

### 4.3 Study Protocol and Measures

Mice were allocated to 5 groups initially matched for body mass: 12AL, 10CR, 20CR, 30CR and 40CR which refers to the percentage level of restriction, i.e., 30CR mice were fed 30% less than their individual total daily calories from baseline. CR was initiated at 5 months of age. Mice were sacrificed with a terminal overdose of CO2 at either 8 months old (after 3 months CR) or 24 months of age (following 19 months CR). From the 64 mice starting the 24‐month study, a total of 33 mice survived to 24 months (Mitchell et al. 2023): 12AL (*n* = 5), 10CR (*n* = 4), 20CR (*n* = 6), 30CR (*n* = 8) and 40CR (*n* = 10). From the 8‐month study only data from the 12AL (*n* = 7) were used for comparison of DNAm at 8 and 24 months of age. Due to multiple analyses on the 8‐month study too few samples were available in the CR groups. Both the 24‐month and 8‐month cohorts, although not large, are of sufficient size to draw meaningful comparative conclusions (Halasz et al. 2024).

Thirteen characteristics were measured for each individual at 24 months of age, i.e., following 19 months of graded CR or 12AL feeding. Methods have previously been detailed for the study of 8‐month‐old mice. Briefly BMR was measured by indirect calorimetry (Mitchell, Delville, Konstantopedos, Derous, et al. 2015). PA and Tb measures were acquired using the VitalView system (Mitchell, Delville, Konstantopedos, Derous, et al. 2015; Mitchell, Delville, et al. 2016). Levels of leptin and insulin were measured in plasma from cardiac punctures taken at time of cull using the mouse metabolic magnetic bead panel (Milliplex MAP Kit; Merck, Germany). IGF was detected using a mouse specific Enzyme Linked Immunoassay (R&D Systems Europe Ltd., UK) (Mitchell, Delville, Konstantopedos, Hurst, et al. 2015). The activity of catalase and SOD was measured in liver homogenates using spectrophotometric assays (Mitchell, Delville, Konstantopedos, Hurst, et al. 2015). Oxidative DNA damage was measured using JAICA Highly Sensitive 8‐OHdG ELISA kit according to manufacturer's instructions (Intra‐inter assay variation: 1%–8.7%). Artificial oxidation of samples was controlled for by extracting DNA using sodium iodide (Mitchell, Delville, Konstantopedos, Hurst, et al. 2015). A necropsy examination was carried out for all mice and disease states, predominantly neoplasia, identified (Mitchell et al. 2023).

### 4.4 Measurement of DNA Methylation

Liver samples were shipped to Zymo Research in DNA Shield (Cat#: R1100, Zymo Research, Irvine, CA). DNA was extracted using Quick‐DNA Magbead Plus Kit (Cat#: D4082) with RNase treatment following the manufacturer's instructions. DNA was finally cleaned up using Genomic DNA Clean & Concentrator‐ 10 (Cat#: D4011).

#### 4.4.1 Library Preparation

The EZ DNA Methylation‐Lightning Kit (Cat#: D5030) following the standard protocol was used for bisulfite conversion. Samples were enriched specifically for the sequencing of > 1000 age‐associated gene loci using Simplified Whole‐panel Amplification Reaction Method (SWARM), where specific CpGs are sequenced at a minimum of 1000X coverage; sequencing was run on Illumina NovaSeq instrument. SWARM is a targeted bisulfite sequencing approach designed to generate reproducible, high‐throughput methylation data. In SWARM, a defined panel of CpG loci within regions of interest (ROI) is profiled in a single‐tube multiplexed reaction. Primers for these loci are designed using Rosefinch, a proprietary bisulfite DNA–specific primer design software developed by Zymo, which ensures specificity for bisulfite‐converted templates.

#### 4.4.2 DNAge Data Analysis

Sequences were identified by Illumina base calling software then aligned to the reference genome using Bismark. Methylation levels for each cytosine were calculated by dividing the number of reads reporting a “c” by the number of reads reporting a “C” or “T”. The percentage of methylation for these specific sequences was used to assess DNA age according to Zymo Research's proprietary DNAge predictor which had been established using elastic net regression to determine the DNAge. The Bedmap program, a component of the BEDOPS suite, was used to annotate mouse clock CpGs using the – echo‐map‐id argument, which outputs IDs of all overlapping mm10 gene annotation elements. Both the clock CpG and mm10 annotation files were formatted as BED files and pre‐sorted using the sort‐bed utility.

### 4.5 Statistical Analysis

Three suites of linear regression were carried out on the DNAm data at each CpG site for 24‐month aged mice: (i) linear regression of DNAm value against graded CR level, (ii) multiple linear regression of the methylation values against all combinations of measured characteristics and CR, so 14 predictor variables in total and (iii) multiple linear regression against all predictor variables after removing the effect of CR on both the predictor variables and DNAm values (we refer to this as the “residuals model”). Following the multiple linear regression analysis, a principal components analysis (PCA) was performed to assess the effects of collinearity between predictor variables. The statistical analysis then looked at whether the directional change in DNAm with increasing CR was consistent with slower ageing of epigenetic state. Statistical tests were carried out using R statistical computing language (R Core Team 2019).

### 4.6 Data Preparation for Linear Regression

The data quality control was similar for all three suites of linear regression. DNAm outliers are commonly prognostic for cancer. Given our focus of DNAm variation with CR, it is apparent that neoplasia is an influential additional variable and to minimise its impact we removed outlier data from our statistical analyses. In consequence of outlier removal, our data satisfies normality requirements for regression and *t*‐test analysis. The approach was to start with the full set of reported DNAm measurements. Then, a linear regression model of DNAm against CR was developed for each CpG site and a 2‐sided Grubbs test (α = 0.05) was applied to the residuals. Outlier data points identified by the Grubbs test were removed from the input data, and the procedure was repeated until either no further outliers were identified or the number of remaining data points at a CpG site was insufficient to perform further analysis. The latter occurred at the few CpG sites that had a large number of measurements that detected zero methylation, typically 20 or more measurements of zero out of 33 individuals. After all outliers were accounted for, then a Shapiro–Wilk test for normal distribution of residuals was applied to ensure that the Grubbs tests had been applied to a data that followed a normal distribution. From the results of the Shapiro–Wilk tests incorporating the Benjamini‐Hochberg procedure (FDR‐adjusted *p* < 0.05) for multiple tests (Benjamini and Hochberg 1995), those CpG sites with extreme non‐normal distribution were removed from the analysis. For multiple linear regression models, the entire process was repeated for each of the thirteen predictor variables in addition to CR, and the resultant fourteen versions of cleansed DNAm input data were combined with all outliers excluded. For the multiple linear regression test starting with residuals, that is, after removing the effect of CR, the procedure was the same apart from starting with residual values rather than the measured DNAm values.

For the thirteen measured characteristic predictor variables both log transformed and untransformed data were evaluated for each variable with the choice being made on the basis of fewest outliers removed and normal distribution of residuals. Eight predictor variable data points were unavailable at the start of analysis: 2 samples for each of PA, Tb and FAA and one sample for Fg and GTT. Seven variables were log transformed: body mass, leptin, insulin, IGF, BMR, PA and Tb. Six variables were not transformed: FAA, catalase, SOD, DNA damage, Fg and GTT. The procedure resulted in 6 of the 421 predictor variable data points being identified as outliers and removed from the analysis so as to avoid the effects of neoplasia and other liver disease on DNAm patterns. For multiple linear regression starting with residuals the effect of CR on the predictor variables was accounted for in a similar manner to DNAm. In this way the residuals models regress residual values of DNAm against residual values for the predictor variables.

### 4.7 Linear Regression Analytical Procedures

Linear regression of DNAm against univariate CR and multiple linear regression models were developed using the fitting linear models (lm) command in R statistical computing language(R Core Team 2019). Following lm runs for multiple linear regression of all possible combinations of predictor variables at each CpG site (16,383 combinations), the lm model with the lowest AIC value was termed the “optimum” model for that CpG site and its properties; *F*‐statistic *p*‐value were carried forward for comparison with “optimum” models at all other CpG sites. This approach was applied to both multiple linear regression models using measured values of DNAm and predictor variables and the models that account for CR and use the residual values as input data (8191 combinations at each CpG site). The *F*‐statistic *p*‐values reported for the “optimum” models across the three suites of linear regression were combined as a single set of tests to allow direct comparison of the relative merits of each type of model at each CpG site. From the combined *p*‐values across all three regression suites, CpG sites that were considered to be significant were selected based on the results of Benjamini‐Hochberg correction for multiple tests (FDR‐adjusted *p* < 0.05). This is a conservative approach which selects fewer significant CpG sites than if the Benjamini‐Hochberg correction was applied to each regression suite separately.

Following the identification of significant CpG sites for linear regression of DNAm against CR an evaluation was carried to determine whether there was a graduated change in DNAm with CR increase. For each significant CpG site a 2‐sided *t*‐test was performed between the DNAm values in the 24‐month 12AL and 40CR. A Benjamini‐Hochberg multiple test (FDR‐adjusted *p* < 0.05) procedure was performed on the *t*‐test results so as to be confident that there was a significant measurable difference between the average DNAm values in the 12AL and 40CR levels. The DNAm values of the 24‐month aged mice 10CR, 20CR and 30CR levels were normalised between 12AL and 40CR for visualisation to evaluate whether there was a graduated change in DNAm with CR change.

Following the multiple linear regression test a PCA analysis was carried out using the measured values of predictor variables to construct orthogonal principal components. For the purpose of maximising the available input to PCA analysis and visualisation the values for the 8 missing and 6 outlier values in the thirteen predictor variables dataset were imputed using the mice (Multivariate Imputation by Chained Equations) package in R (van Buuren and Groothuis‐Oudshoorn 2011). A multiple linear regression using the principal components and the cleansed DNAm data was carried out and comparison was made of highly significant terms in the optimum models using measured predictor variables and PCs. The graphical presentation of PCA results used the ggplot package in R (Wickham 2009).

### 4.8 Epigenetic Ageing

In addition to the dataset for the 33 24‐month‐old mice, DNAm data was also available for a group of 7 8‐month‐old 12AL mice. Epigenetic ageing was assessed through comparison of DNAm measurements for the 24‐month 12AL, 24‐month 40CR and 8‐month 12AL levels. The underlying assumption in assessing epigenetic ageing was that a DNAm change between 24‐month 12AL and 24‐month 40CR levels that occurred in the same direction as the change in DNAm 24‐month 12AL and 8‐month 12AL levels was indicative of slowing of epigenetic ageing. The starting point was to select the CpG sites which had significant separation between the average DNAm values for 24‐month 12AL and 24‐month 40CR, that is, those sites used in the graduated response to CR analysis. A further *t*‐test with Benjamini‐Hochberg multiple test (FDR‐adjusted *p* < 0.05) procedure was carried out for 24‐month 12AL and 8‐month 12AL levels to ensure that a statistically significant difference existed between the average DNAm values of the two 12AL groups. The ratio of DNAm changes due to CR effects and those of different aged mice together with the direction of change provided results for visualisation of slowing of epigenetic ageing.

## 5 Limitations

Our study only involved male mice. Given recent work suggesting sex differences in CR responses, understanding if the same patterns are observed in females will be a key future goal. Our standard bisulfite sequencing methods are unable to distinguish between methylation (5mC) and hydroxymethylation (5hmC); our methylation value is the sum of 5mC and 5hmC. Although we showed that CR caused slowing of epigenetic ageing, we did not elucidate the mechanism by which CR results in the epigenetic changes we observed. Mechanistic studies and functional confirmation would require additional knockdown or overexpression studies. While such experiments are important, they are beyond the scope of the current study. We only used a single epigenetic clock. Other clocks have been developed but evaluate DNAm changes at different CpG sites, and we do not know whether the same slowing of epigenetic ageing with CR would be observed with these other tools. Finally, we did not discover how these epigenetic changes might potentially underpin changed gene expression profiles and the life‐extending impacts of CR.

## Author Contributions

S.E.M. performed all experimental animal work and assays related to individual characteristics. X.Y., W.G. and E.C. carried out the DNAm analysis. T.P.M. analysed the data, prepared all figures and drafted the manuscript. J.R.S. and S.E.M. reviewed and edited the manuscript. All authors approved the final version.

## Funding

The funding was raised by J.R.S. Biotechnology and Biological Sciences Research Council (Grant BB/G009953/1).

## Conflicts of Interest

The authors declare no conflicts of interest.

## Supporting information


**Appendix S1:** acel70342‐sup‐0001‐AppendixS1.docx.

## Data Availability

The data that support the findings of this study will be uploaded to our Open Science Framework for ‘The graded Calorie Restriction project’, https://doi.org/10.17605/OSF.IO/9YATH.
