# Supplementary material for: Graded Calorie Restriction Causes Graded Slowing of Epigenetic Ageing in Mice
Source: Aging Cell. 2025 Dec 23;25(1):e70342. doi: 10.1111/acel.70342 (PMC12724011; doi:10.1111/acel.70342)
Supplement: Supplementary file 1 — Appendix S1: acel70342‐sup‐0001‐AppendixS1.docx. [file ACEL-25-e70342-s001.docx]

**Supplementary Material**

**Supplementary Material Table 1**. Measurements on male C57BL/6J mice aged 24 months following 19 months graded calorie restriction. These measurements were used as predictor variables in regression analyses to determine whether they were related to changes in DNA methylation. The variables measured were calorie restriction level (CR), DNAge®, cull body mass; circulating leptin, insulin, and insulin like growth factor-1 (IGF); basal metabolic rate (BMR); physical activity (PA), body temperature (Tb), food anticipatory activity (FAA); catalase, and superoxide dismutase (SOD) antioxidant activity, DNA damage in the liver, fasting glucose level (Fg) and in the area under the curve from glucose tolerance test (GTT).

**Supplementary Material Table 2.**

**Supplementary Material Table 3**. Genes with three or more significant CpG sites associated with liver disease or liver function

| **Genes (synonyms) with role in hepatocellular carcinoma (HCC) promotion or suppression** | | |
| --- | --- | --- |
| Dnajb2 | High expression is unfavourable in HCC | The Human Genome Atlas. Expression of DNAJB2 in liver cancer. https://www.proteinatlas.org/ENSG00000135924-DNAJB2/cancer/liver+cancer#LIHC_TCGA (2025)  Uhlén, M. et al. Tissue-based map of the human proteome. Science 347, 1260419 (2015) |
| Fam84b (Lratd2) | Collaborates with MyC and POU5F1B in HCC proliferation | Gu, Y. et al. The Oncogenic Potential of the Centromeric Border Protein FAM84B of the 8q24.21 Gene Desert. Genes 11, 312 (2020)  Pan, Y. et al. POU5F1B promotes hepatocellular carcinoma proliferation by activating AKT. Biomed. Pharmacother. 100, 374–380 (2018) |
| Hdgfl2 (Hrp2) | Promotes cell growth in HCC | Gao, K. et al. HDGF-related protein-2 (HRP-2) acts as an oncogene to promote cell growth in hepatocellular carcinoma. Biochem. Biophys. Res. Commun. 458, 849–855 (2015) |
| Lncppara | Facilitates HCC | Xia, Y. et al. MIRLET7BHG promotes hepatocellular carcinoma progression by activating hepatic stellate cells through exosomal SMO to trigger Hedgehog pathway. Cell Death Dis. 12, 1–17 (2021) |
| Nlrp6 | Conflicting repots: Tumor suppression in HCC (Gao et al) versus tumor promotion (Li et al). | NLRP6 potential tumor suppressor gene (Gao et al, Cancer medicine, 2023). NLRP6 promotes HCC progression by inhibiting macrophage infiltration and suppressing phagocytosis (Li et al, Gut, 2025). |
| Qprt | Enhances cancer invasiveness for breast cancer and similar mechanisms observed in HCC | Khalid, M. et al. Carcinoma-specific expression of P2Y11 receptor and its contribution in ATP-induced purinergic signalling and cell migration in human hepatocellular carcinoma cells. Oncotarget 8, 37278–37290 (2017)  Liu, C.-L. et al. Quinolinate Phosphoribosyltransferase Promotes Invasiveness of Breast Cancer Through Myosin Light Chain Phosphorylation. Front. Endocrinol. 11, (2021) |
| Wasf3 (Wave3) | A master regulator for metastatic signalling. Promotes HCC invasiveness and metastasis. Hypomethylation is a published biomarker for HCC | Ji, Y. et al. Overexpression of WAVE3 promotes tumor invasiveness and confers an unfavorable prognosis in human hepatocellular carcinoma. Biomed. Pharmacother. 69, 409–415 (2015)  Loveless, R. & Teng, Y. Targeting WASF3 Signaling in Metastatic Cancer. Int. J. Mol. Sci. 22, 836 (2021)  Ahn, H.R., Baek, G.O., Yoon, M.G., Son, J.A., Yoon, J.H., Cheong, J.Y., Cho, H.J., Kang, H.C., Eun, J.W., Kim, S.S., 2022. Hypomethylation-mediated upregulation of the WASF2 promoter region correlates with poor clinical outcomes in hepatocellular carcinoma. J. Exp. Clin. Cancer Res. 41, 158. https://doi.org/10.1186/s13046-022-02365-7 |
| Zic1 | HCC tumor suppressor, hypermethylation is correlated with tumor size and a published biomarker for HCC | Wang, Y.-Y. et al. Role of ZIC1 methylation in hepatocellular carcinoma and its clinical significance. Tumor Biol. 35, 7429–7433 (2014)  Wang, Y.-Y., Jiang, J.-X., Ma, H., Han, J., Sun, Z.-Y., Liu, Z.-M., Xu, Z.-G., 2014. Role of ZIC1 methylation in hepatocellular carcinoma and its clinical significance. Tumor Biol. 35, 7429–7433. https://doi.org/10.1007/s13277-014-1971-4 |
| Clvs2 | Upregulated in Clonorchis sinensis infected, and lenvatinib resistant HCC | Chen, J. et al. Clonorchis sinensis-infected hepatocellular carcinoma exhibits distinct tumor microenvironment and molecular features. Front. Immunol. 16, (2025)  Zhang, P. et al. GALNT6 drives lenvatinib resistance in hepatocellular carcinoma through autophagy and cancer-associated fibroblast activation. Cell. Oncol. 47, 2439–2460 (2024) |
| 1010001N08Rik (LncGata6) | Activates Ets homologous factor (EHF), and EHF knockdown decreases liver metastasis of colorectal carcinoma cells | Jung, Y.-S., Kim, M. J. & Park, J.-I. LncGata6-controlled stemness in regeneration and cancer. Non-Coding RNA Investig. 3, 4 (2019)  Wang, L. et al. EHF promotes colorectal carcinoma progression by activating TGF-β1 transcription and canonical TGF-β signaling. Cancer Sci. 111, 2310–2324 (2020) |
| Calb2 | Activates signalling pathway to promote HCC metastasis | Chu, H. et al. All-Ion Monitoring-Directed Low-Abundance Protein Quantification Reveals CALB2 as a Key Promoter in Hepatocellular Carcinoma Metastasis. Anal. Chem. 94, 6102–6111 (2022)  Tao, J. et al. CALB2 drives pancreatic cancer metastasis through inflammatory reprogramming of the tumor microenvironment. J. Exp. Clin. Cancer Res. 43, 277 (2024) |
| Itgb4 | Mediates the pathway to promote HCC metastasis. ERBB receptors are linked to human cancer pathogenesis | Arteaga, C. L. & Engelman, J. A. ERBB receptors: From oncogene discovery to basic science to mechanism-based cancer therapeutics. Cancer Cell 25, 282–303 (2014)  Shi, W.-K., Shang, Q.-L. & Zhao, Y.-F. SPC25 promotes hepatocellular carcinoma metastasis via activating the FAK/PI3K/AKT signaling pathway through ITGB4. Oncol. Rep. 47, 1–14 (2022) |
| Mbd2 | Regulates transcription onset of HCC critical genes and induces invasiveness | Stefanska, B. et al. Transcription onset of genes critical in liver carcinogenesis is epigenetically regulated by methylated DNA-binding protein MBD2. Carcinogenesis 34, 2738–2749 (2013) |
| **Genes with role in other liver role in disease, protection or cell proliferation** | | |
| Tnip1 | Protects hepatic cell injury through inhibition of pro-inflammatory cytokines | Zhang, Y. et al. TNIP1 alleviates hepatic ischemia/reperfusion injury via the TLR2-Myd88 pathway. Biochem. Biophys. Res. Commun. 501, 186–192 (2018) |
| Nfic | Regulate hepatocyte proliferation during liver regeneration and an antioncogene for lung squamous cell carcinoma | Edelmann, S. et al. Nuclear Factor I-C acts as a regulator of hepatocyte proliferation at the onset of liver regeneration. Liver Int. 35, 1185–1194 (2015)  Zhang, H. et al. Transcription factor NFIC functions as a tumor suppressor in lung squamous cell carcinoma progression by modulating lncRNA CASC2. Cell Cycle 21, 63–73 (2022) |
| Rbpms2 | Overexpressed in liver cells resistant to Hepatitis C antiviral drug Ribavirin. In other tissue, downregulation promotes GC | Cheng, M. et al. DNA methylation of RNA-binding protein for multiple splicing 2 functions as diagnosis biomarker in gastric cancer pathogenesis and its potential clinical significance. Bioengineered 13, 4347–4360 (2022)  Satoh, S. et al. Establishment of Hepatitis C Virus RNA-Replicating Cell Lines Possessing Ribavirin-Resistant Phenotype. PLOS ONE 10, e0118313 (2015) |
| Iffo2 | Differentially expressed in significant liver fibrosis | Johnson, K. et al. Increased serum miR-193a-5p during non-alcoholic fatty liver disease progression: Diagnostic and mechanistic relevance. JHEP Rep. 4, 100409 (2022) |
| **Genes with no information specific to liver function** | | |
| Map10 | Regulation of cell division and promotes microtubule stability | The Human Protein Atlas. MAP10 protein expression summary. https://www.proteinatlas.org/ENSG00000212916-MAP10 (2025) |
| Actl7b | An actin-related protein. ARPs involved in diverse cellular processes, including vesicular transport, spindle orientation, nuclear migration and chromatin remodeling | Clement, T. M. et al. Actin-related protein ACTL7B ablation leads to OAT with multiple morphological abnormalities of the flagellum and male infertility in mice†. Biol. Reprod. 108, 447–464 (2023) |
| Gm43401 | No information |  |

**Supplementary Material Table 4**. Genes with three or more significant CpG sites links to cancer

**Genes identified through univariate linear regression that act to suppress or mitigate cancer:**

| Gene | Association with Cancer and References |
| --- | --- |
| Dnajb2 | A heat shock protein with key role in preventing misfolded protein aggregation and degradation and removal of the protein aggregates. Upregulated in human skeletal muscle during recovery from damage (Chen et al., 2023; Claeys et al., 2010; Howarth et al., 2007; Stelzer et al., 2016) and downregulated in cancer cells (Sterrenberg et al., 2011). |
| Map10 | Human synonyms KIAA1383 and MTR120. Promotes microtubular stability and normal cytokinesis with low level of MAP10 mRNA linked with several cancers (Fong et al., 2013; Trasierras et al., 2022). |
| Nfic | A tumor suppressor in lung squamous cell carcinoma tumorigenesis |
| Nlrp6 | A tumor suppressor in human gastric cancer, reduced Nlrp6 expression speeds up hGC progression, and in hematopoietic cells Nlrp6 is critical for protection against inflammation-related colon tumorigenesis (Bai and Li, 2020; Chen et al., 2011; Wang et al., 2018). However, in hepatocellular carcinoma Gao et al (2023) report tumor suppression due to higher immune cell infiltration but Li et la (2025) report tumor promotion due to inhibiting macrophage infiltration and supressing phagocytosis. |
| Rbpms2 | Overexpression of Rbpms2 inhibits human gastric cancer, increased DNAm being associated with tumor invasion, shorter survival and aging (Ananthamohan et al., 2024; Cheng et al., 2022; Peng et al., 2020). |
| Zic1 | A tumor suppressor which is silenced through promoter hypermethylation in hGC and hepatocellular carcinoma (Wang et al., 2009, 2014). |

**Genes identified through univariate linear regression associated with tumorigenesis and cancer progression**

| Gene | Association with Cancer and References |
| --- | --- |
| Clvs2 | An oncogene for colon adenocarcinoma (Bai et al., 2024). Clvs2 functions in a pathway between early endosome and mature lysosome. Endosomes are involved in cell proliferation, metabolism and signalling and lysosomes regulate cell adhesion and invasion; overexpression of either organelle may cause carcinogenesis (Jeger, 2020; Katoh et al., 2009). Expression is highly correlated with prostate cancer (Zheng et al., 2023). |
| Fam84b | Correlates with tumorigenesis and tumor progression in breast, pancreatic, gastric, prostate and oesophageal cancers (Canisius et al., 2022; Gu et al., 2020). Various pathways including alterations to DNA repair mechanisms (Arroyo et al., 2015); association with Myc with which Fam84b is co-amplified in over 20 cancer types (Gu et al., 2020; Homer-Bouthiette et al., 2018); interacts with nucleophosmin to suppress cyclin-dependent kinase inhibitor 2A protein expression (Wang et al., 2022); and promoting tumorigenesis through the activation of the NF-κB and death receptor signalling pathways (Zhang and Yang, 2023). |
| Hdgfl2 | Promotes cell growth in hepatocellular carcinoma and in colorectal cancer higher expression level of Hdgfl2 in tumor tissue leads to a worse survival prognosis (El Hindi et al., 2024; Gao et al., 2015). |
| Qprt | (El Hindi et al., 2024; Gao et al., 2015). Qprt is an anti-apoptosis gene and key enzyme in de novo nicotinamide adenine dinucleotide (NAD^+^) synthesis; the gene product inhibits spontaneous cell death, enhances breast cancer and has higher than normal expression in many cancer tissues (Ishidoh et al., 2010; Liu et al., 2021). |
| Wasf3 | A master regulator that controls many components of metastatic signalling complexes (Loveless and Teng, 2021; Qin et al., 2019). Through demethylation of its promoter Wasf3 drives breast cancer metastasis. |

**Genes identified through multiple linear regression after accounting for the effects of CR on DNAm and predictor variables**

| Gene (Cancer Role) | Predictor  Variable(s) | Association with Cancer and References |
| --- | --- | --- |
| 1010001N08Rik  (Promote) | Ln(BMR) Catalase | Also called Gata6os, Gata6as and LncGata6 (Mouse Genome Informatics, 2024). A long non-coding RNA gene. Exerts control by recruiting chromatin modifiers with a key role in gene regulation (Flynn and Chang, 2014; Statello et al., 2021). Highly expressed in colorectal cancer stem cells and promotes tumorigenesis and cancer progression (Jung et al., 2019; Zhu et al., 2018). Potential therapeutic target as cancerous intestinal stem cells deficient in 1010001N08Rik lose fitness (Hatzis and Snippert, 2018). |
| Iffo2  (Suppress) | GTT  Ln(Leptin)  Ln(Mass) | Protein product is a protective prognostic factor for head and neck squamous (Li et al., 2023) and substantially downregulated in cutaneous squamous cancer (Tsang et al., 2023). Methylation associated with dietary glycaemic load (Ott et al., 2023) |
| Itgb4  (Promote) | GTT | Itgb4 is pivotal for invasive carcinomas and expression has been correlated with many cancers (Huang et al., 2021) and its role demonstrated through immunologic strategies to target Itgb4 that inhibited tumor growth and reduced secondary-tumor initiating capacity (Ruan et al., 2020). The role of Itgb4 protein is to enhance cancer metabolism by triggering aerobic glycolysis in cancer-associated fibroblasts which stimulate angiogenesis, tumor formation and metastasis (Sung et al., 2020). In prostate cancer altered methylation of Itgb4 was found at different stages of tumor progression (Wilkinson et al., 2020). Upregulated in diabetic glomeruli (Levin et al., 2020) |
| Mbd2  (Promote) | Insulin | Mbd2 codes Methyl-CpG Binding Domain Protein 2 which contains a methyl-CpG-binding domain capable of binding to methylated DNA and is an integral part of the Nucleosome Remodeling Deacetylase complex (NuRD) complex. Mbd2 occupies methylated CpG island-containing promoters of inactive genes and is an essential regulator of immune function and tumorigenesis (Hainer et al., 2016; Wood and Zhou, 2016). Elevated Mbd2 is associated with invasion and metastasis of lung adenocarcinoma cells through a mechanism where Mbd2 selectively binds to methylated CpG DNA within the damage-specific DNA binding protein 2 (DDB2) promoter which is itself a cancer suppressor (Cui et al., 2018; Zhang et al., 2023). Elevated Mbd2 expression is also associated with type-2 diabetes mellitus (T2DM) and leads to a general dysregulation of DNAm (Karachanak-Yankova et al., 2015). |
| Tnip1  (Tissue dependent) | Insulin | Associated with cancer and T2DM. A tumor inhibitor of human clear cell renal cell carcinomas but for glioma, a malignant tumor of the central nervous system, elevated Tnip1 is associated with poor prognosis (Lei et al., 2020; Yang et al., 2019). Associated with T2DM (Zhou et al., 2023). |

**References for Supplementary Table 4**

Ananthamohan, K., Stelzer, J.E., Sadayappan, S., 2024. Hypertrophic cardiomyopathy in MYBPC3 carriers in aging. J. Cardiovasc. Aging 4. https://doi.org/10.20517/jca.2023.29

Arroyo, R., Suñé, G., Zanzoni, A., Duran-Frigola, M., Alcalde, V., Stracker, T.H., Soler-López, M., Aloy, P., 2015. Systematic Identification of Molecular Links between Core and Candidate Genes in Breast Cancer. J. Mol. Biol. 427, 1436–1450. https://doi.org/10.1016/j.jmb.2015.01.014

Bai, H., Yan, D.-S., Chen, Y.-L., Li, Q.-Z., Qi, Y.-C., 2024. Potential biomarkers: The hypomethylation of cg18949415 and cg22193385 sites in colon adenocarcinoma. Comput. Biol. Med. 169, 107884. https://doi.org/10.1016/j.compbiomed.2023.107884

Bai, Y., Li, S., 2020. Long noncoding RNA OIP5-AS1 aggravates cell proliferation, migration in gastric cancer by epigenetically silencing NLRP6 expression via binding EZH2. J. Cell. Biochem. 121, 353–362. https://doi.org/10.1002/jcb.29183

Canisius, J., Wagner, A., Bunk, E.C., Spille, D.C., Stögbauer, L., Grauer, O., Hess, K., Thomas, C., Paulus, W., Stummer, W., Senner, V., Brokinkel, B., 2022. Expression of decitabine-targeted oncogenes in meningiomas in vivo. Neurosurg. Rev. 45, 2767–2775. https://doi.org/10.1007/s10143-022-01789-1

Chen, G.Y., Liu, M., Wang, F., Bertin, J., Núñez, G., 2011. A Functional Role for Nlrp6 in Intestinal Inflammation and Tumorigenesis. J. Immunol. 186, 7187–7194. https://doi.org/10.4049/jimmunol.1100412

Chen, H., Lin, C., Xue, H.-M., Chen, C., Yang, M., 2023. The heat shock protein DNAJB2 as a novel biomarker for essential thrombocythemia diagnosis associated with immune infiltration. Thromb. Res. 223, 131–138. https://doi.org/10.1016/j.thromres.2023.01.029

Cheng, M., Zhan, X., Xu, Y., Wang, S., Zhang, H., Fang, L., Jin, H., Chen, W., 2022. DNA methylation of RNA-binding protein for multiple splicing 2 functions as diagnosis biomarker in gastric cancer pathogenesis and its potential clinical significance. Bioengineered 13, 4347–4360. https://doi.org/10.1080/21655979.2022.2032965

Claeys, K.G., Sozanska, M., Martin, J.-J., Lacene, E., Vignaud, L., Stockholm, D., Laforêt, P., Eymard, B., Kichler, A., Scherman, D., Voit, T., Israeli, D., 2010. DNAJB2 Expression in Normal and Diseased Human and Mouse Skeletal Muscle. Am. J. Pathol. 176, 2901–2910. https://doi.org/10.2353/ajpath.2010.090663

Cui, T., Srivastava, A.K., Han, C., Wu, D., Wani, N., Liu, L., Gao, Z., Qu, M., Zou, N., Zhang, X., Yi, P., Yu, J., Bell, E.H., Yang, S.-M., Maloney, D.J., Zheng, Y., Wani, A.A., Wang, Q.-E., 2018. DDB2 represses ovarian cancer cell dedifferentiation by suppressing ALDH1A1. Cell Death Dis. 9, 1–15. https://doi.org/10.1038/s41419-018-0585-y

El Hindi, K., Brachtendorf, S., Hartel, J.C., Renné, C., Birod, K., Schilling, K., Labocha, S., Thomas, D., Ferreirós, N., Hahnefeld, L., Dorochow, E., Del Turco, D., Deller, T., Scholich, K., Fuhrmann, D.C., Weigert, A., Brüne, B., Geisslinger, G., Wittig, I., Link, K.-H., Grösch, S., 2024. Hypoxia induced deregulation of sphingolipids in colon cancer is a prognostic marker for patient outcome. Biochim. Biophys. Acta BBA - Mol. Basis Dis. 1870, 166906. https://doi.org/10.1016/j.bbadis.2023.166906

Flynn, R.A., Chang, H.Y., 2014. Long Noncoding RNAs in Cell-Fate Programming and Reprogramming. Cell Stem Cell 14, 752–761. https://doi.org/10.1016/j.stem.2014.05.014

Fong, K., Leung, J.W., Li, Y., Wang, W., Feng, L., Ma, W., Liu, D., Songyang, Z., Chen, J., 2013. MTR120/KIAA1383, a novel microtubule-associated protein, promotes microtubule stability and ensures cytokinesis. J. Cell Sci. 126, 825–837. https://doi.org/10.1242/jcs.116137

Gao, K., Xu, C., Jin, X., Wumaier, R., Ma, J., Peng, J., Wang, Y., Tang, Y., Yu, L., Zhang, P., 2015. HDGF-related protein-2 (HRP-2) acts as an oncogene to promote cell growth in hepatocellular carcinoma. Biochem. Biophys. Res. Commun. 458, 849–855. https://doi.org/10.1016/j.bbrc.2015.02.042

Gu, Y., Lin, X., Kapoor, A., Chow, M.J., Jiang, Y., Zhao, K., Tang, D., 2020. The Oncogenic Potential of the Centromeric Border Protein FAM84B of the 8q24.21 Gene Desert. Genes 11, 312. https://doi.org/10.3390/genes11030312

Hainer, S.J., McCannell, K.N., Yu, J., Ee, L.-S., Zhu, L.J., Rando, O.J., Fazzio, T.G., 2016. DNA methylation directs genomic localization of Mbd2 and Mbd3 in embryonic stem cells. eLife 5, e21964. https://doi.org/10.7554/eLife.21964

Hatzis, P., Snippert, H.J.G., 2018. Long noncoding RNAs in gut stem cells. Nat. Cell Biol. 20, 1106–1107. https://doi.org/10.1038/s41556-018-0208-y

Homer-Bouthiette, C., Zhao, Y., Shunkwiler, L.B., Van Peel, B., Garrett-Mayer, E., Baird, R.C., Rissman, A.I., Guest, S.T., Ethier, S.P., John, M.C., Powers, P.A., Haag, J.D., Gould, M.N., Smits, B.M.G., 2018. Deletion of the murine ortholog of the 8q24 gene desert has anti-cancer effects in transgenic mammary cancer models. BMC Cancer 18, 1233. https://doi.org/10.1186/s12885-018-5109-8

Howarth, J., Kelly, S., Keasey, M., Glover, C., Lee, Y.-B., Mitrophanous, K., Chapple, J., Gallo, J., Cheetham, M., Uney, J., 2007. Hsp40 Molecules That Target to the Ubiquitin-proteasome System Decrease Inclusion Formation in Models of Polyglutamine Disease. Mol. Ther. 15, 1100–1105. https://doi.org/10.1038/sj.mt.6300163

Huang, W., Fan, L., Tang, Y., Chi, Y., Li, J., 2021. A Pan-Cancer Analysis of the Oncogenic Role of Integrin Beta4 (ITGB4) in Human Tumors. Int. J. Gen. Med. 14, 9629–9645. https://doi.org/10.2147/IJGM.S341076

Ishidoh, K., Kamemura, N., Imagawa, T., Oda, M., Sakurai, J., Katunuma, N., 2010. Quinolinate phosphoribosyl transferase, a key enzyme in de novo NAD(+) synthesis, suppresses spontaneous cell death by inhibiting overproduction of active-caspase-3. Biochim. Biophys. Acta 1803, 527–533. https://doi.org/10.1016/j.bbamcr.2010.02.007

Jeger, J.L., 2020. Endosomes, lysosomes, and the role of endosomal and lysosomal biogenesis in cancer development. Mol. Biol. Rep. 47, 9801–9810. https://doi.org/10.1007/s11033-020-05993-4

Jung, Y.-S., Kim, M.J., Park, J.-I., 2019. LncGata6-controlled stemness in regeneration and cancer. Non-Coding RNA Investig. 3, 4. https://doi.org/10.21037/ncri.2019.01.02

Karachanak-Yankova, S., Dimova, R., Nikolova, D., Nesheva, D., Koprinarova, M., Maslyankov, S., Tafradjiska, R., Gateva, P., Velizarova, M., Hammoudeh, Z., Stoynev, N., Toncheva, D., Tankova, T., Dimova, I., 2015. Epigenetic alterations in patients with type 2 diabetes mellitus. Balk. J. Med. Genet. 18, 15–24.

Katoh, Y., Ritter, B., Gaffry, T., Blondeau, F., Höning, S., McPherson, P.S., 2009. The Clavesin Family, Neuron-specific Lipid- and Clathrin-binding Sec14 Proteins Regulating Lysosomal Morphology *. J. Biol. Chem. 284, 27646–27654. https://doi.org/10.1074/jbc.M109.034884

Lei, Q., Gu, H., Li, L., Wu, T., Xie, W., Li, M., Zhao, N., 2020. TNIP1-mediated TNF-α/NF-κB signalling cascade sustains glioma cell proliferation. J. Cell. Mol. Med. 24, 530–538. <https://doi.org/10.1111/jcmm.14760>

Levin, A., Reznichenko, A., Witasp, A., Liu, P., Greasley, P.J., Sorrentino, A., Blondal, T., Zambrano, S., Nordström, J., Bruchfeld, A., Barany, P., Ebefors, K., Erlandsson, F., Patrakka, J., Stenvinkel, P., Nyström, J., Wernerson, A., 2020. Novel insights into the disease transcriptome of human diabetic glomeruli and tubulointerstitium. Nephrol. Dial. Transplant. 35, 2059–2072. https://doi.org/10.1093/ndt/gfaa121

Li, S., Fu, Y., Jia, X., Liu, Z., Qian, Z., Zha, H., Lei, G., Yu, L., Zhang, X., Zhang, Ting, Zhang, Tianyi, Han, J., Shi, Y., Safadi, R., Lu, Y., 2025. NLRP6 deficiency enhances macrophage-mediated phagocytosis via E-Syt1 to inhibit hepatocellular carcinoma

Li, Z., Zheng, C., Liu, H., Lv, J., Wang, Y., Zhang, K., Kong, S., Chen, F., Kong, Y., Yang, X., Cheng, Y., Yang, Z., Zhang, C., Tian, Y., 2023. A novel oxidative stress-related gene signature as an indicator of prognosis and immunotherapy responses in HNSCC. Aging 15, 14957–14984. https://doi.org/10.18632/aging.205323

Liu, C.-L., Cheng, S.-P., Chen, M.-J., Lin, C.-H., Chen, S.-N., Kuo, Y.-H., Chang, Y.-C., 2021. Quinolinate Phosphoribosyltransferase Promotes Invasiveness of Breast Cancer Through Myosin Light Chain Phosphorylation. Front. Endocrinol. 11.

Loveless, R., Teng, Y., 2021. Targeting WASF3 Signaling in Metastatic Cancer. Int. J. Mol. Sci. 22, 836. https://doi.org/10.3390/ijms22020836

Mouse Genome Informatics, 2024. Gata6os MGI Mouse Gene Detail - MGI:1915684 - GATA binding protein 6, opposite strand [WWW Document]. Mouse Genome Inform. URL https://www.informatics.jax.org/marker/MGI:1915684 (accessed 8.8.24).

Ott, R., Stein, R., Hauta-alus, H.H., Ronkainen, J., Fernández-Barrés, S., Spielau, U., Kirsten, H., Poulain, T., Melton, P.E., Küpers, L.K., Azaryah, H., Colombo, M., Landgraf, K., Tobi, E.W., O’Sullivan, T., Huang, R.-C., Campoy, C., Winkler, C., Vioque, J., Vrijheid, M., Kiess, W., Körner, A., Sebert, S., Jarvelin, M.-R., Ziegler, A.-G., Hummel, S., 2023. Epigenome-Wide Meta-analysis Reveals Associations Between Dietary Glycemic Index and Glycemic Load and DNA Methylation in Children and Adolescents of Different Body Sizes. Diabetes Care 46, 2067–2075. <https://doi.org/10.2337/dc23-0474>

Peng, Y., Wu, Q., Wang, L., Wang, H., Yin, F., 2020. A DNA methylation signature to improve survival prediction of gastric cancer. Clin. Epigenetics 12, 15. https://doi.org/10.1186/s13148-020-0807-x

Qin, H., Lu, S., Thangaraju, M., Cowell, J.K., 2019. Wasf3 Deficiency Reveals Involvement in Metastasis in a Mouse Model of Breast Cancer. Am. J. Pathol. 189, 2450–2458. https://doi.org/10.1016/j.ajpath.2019.08.012

Ruan, S., Lin, M., Zhu, Y., Lum, L., Thakur, A., Jin, R., Shao, W., Zhang, Y., Hu, Y., Huang, S., Hurt, E.M., Chang, A.E., Wicha, M.S., Li, Q., 2020. Integrin β4–Targeted Cancer Immunotherapies Inhibit Tumor Growth and Decrease Metastasis. Cancer Res. 80, 771–783. https://doi.org/10.1158/0008-5472.CAN-19-1145

Statello, L., Guo, C.-J., Chen, L.-L., Huarte, M., 2021. Gene regulation by long non-coding RNAs and its biological functions. Nat. Rev. Mol. Cell Biol. 22, 96–118. https://doi.org/10.1038/s41580-020-00315-9

Stelzer, G., Rosen, N., Plaschkes, I., Zimmerman, S., Twik, M., Fishilevich, S., Stein, T.I., Nudel, R., Lieder, I., Mazor, Y., Kaplan, S., Dahary, D., Warshawsky, D., Guan-Golan, Y., Kohn, A., Rappaport, N., Safran, M., Lancet, D., 2016. The GeneCards Suite: From Gene Data Mining to Disease Genome Sequence Analyses. Curr. Protoc. Bioinforma. 54, 1.30.1-1.30.33. https://doi.org/10.1002/cpbi.5

Sterrenberg, J.N., Blatch, G.L., Edkins, A.L., 2011. Human DNAJ in cancer and stem cells. Cancer Lett. 312, 129–142. https://doi.org/10.1016/j.canlet.2011.08.019

Sung, J.S., Kang, C.W., Kang, S., Jang, Y., Chae, Y.C., Kim, B.G., Cho, N.H., 2020. ITGB4-mediated metabolic reprogramming of cancer-associated fibroblasts. Oncogene 39, 664–676. https://doi.org/10.1038/s41388-019-1014-0

Trasierras, A.M., Luna, J.M., Ventura, S., 2022. Improving the understanding of cancer in a descriptive way: An emerging pattern mining-based approach. Int. J. Intell. Syst. 37, 2822–2848. https://doi.org/10.1002/int.22503

Tsang, D.A., Tam, S.Y.C., Oh, C.C., 2023. Molecular Alterations in Cutaneous Squamous Cell Carcinoma in Immunocompetent and Immunosuppressed Hosts—A Systematic Review. Cancers 15, 1832. https://doi.org/10.3390/cancers15061832

Wang, F., Cheng, C., Wang, X., Chen, F., Li, H., Zhou, Y., Wang, Y., Hu, X., Kong, P., Zhang, L., Cheng, X., Cui, Y., 2022. Elevated FAM84B promotes cell proliferation via interacting with NPM1 in esophageal squamous cell carcinoma. Cell Death Discov. 8, 1–12. https://doi.org/10.1038/s41420-022-00984-9

Wang, L.J., Jin, H.C., Wang, X., Lam, E.K.Y., Zhang, J.B., Liu, X., Chan, F.K.L., Si, J.M., Sung, J.J.Y., 2009. ZIC1 is downregulated through promoter hypermethylation in gastric cancer. Biochem. Biophys. Res. Commun. 379, 959–963. https://doi.org/10.1016/j.bbrc.2008.12.180

Wang, Q., Wang, C., Chen, J., 2018. NLRP6, decreased in gastric cancer, suppresses tumorigenicity of gastric cancer cells. Cancer Manag. Res. 10, 6431–6444. https://doi.org/10.2147/CMAR.S182980

Wang, Y.-Y., Jiang, J.-X., Ma, H., Han, J., Sun, Z.-Y., Liu, Z.-M., Xu, Z.-G., 2014. Role of ZIC1 methylation in hepatocellular carcinoma and its clinical significance. Tumor Biol. 35, 7429–7433. https://doi.org/10.1007/s13277-014-1971-4

Wilkinson, E.J., Woodworth, A.M., Parker, M., Phillips, J.L., Malley, R.C., Dickinson, J.L., Holloway, A.F., 2020. Epigenetic regulation of the ITGB4 gene in prostate cancer. Exp. Cell Res. 392, 112055. https://doi.org/10.1016/j.yexcr.2020.112055

Wood, K.H., Zhou, Z., 2016. Emerging Molecular and Biological Functions of MBD2, a Reader of DNA Methylation. Front. Genet. 7.

Yang, Y., Fan, J., Han, S., Li, E., 2019. TNIP1 Inhibits Proliferation And Promotes Apoptosis In Clear Cell Renal Carcinoma Through Targeting C/Ebpβ. OncoTargets Ther. 12, 9861–9871. https://doi.org/10.2147/OTT.S216138

Zhang, L., Wang, S., Wu, G.-R., Yue, H., Dong, R., Zhang, S., Yu, Q., Yang, P., Zhao, J., Zhang, H., Yu, J., Yuan, X., Xiong, W., Yang, X., Yong, T., Wang, C.-Y., 2023. MBD2 facilitates tumor metastasis by mitigating DDB2 expression. Cell Death Dis. 14, 1–11. https://doi.org/10.1038/s41419-023-05804-1

Zhang, Y., Yang, F., 2023. FAM84B promotes breast cancer tumorigenesis through activation of the NF-KB and death receptor signaling pathways. Pathol. Res. Pract. 249, 154785. https://doi.org/10.1016/j.prp.2023.154785

Zheng, Y., Wang, Y., He, H., Zou, Z., Lu, H., Li, J., Cai, J., Wang, K., 2023. Transcriptome Data Reveal Geographic Heterogeneity in Gene Expression in Patients with Prostate Cancer. Rep. Biochem. Mol. Biol. 12, 92–101. <https://doi.org/10.52547/rbmb.12.1.92>

Zhou, C., She, X., Gu, C., Hu, Y., Ma, M., Qiu, Q., Sun, T., Xu, X., Chen, H., Zheng, Z., 2023. FTO fuels diabetes-induced vascular endothelial dysfunction associated with inflammation by erasing m6A methylation of TNIP1. J. Clin. Invest. 133. https://doi.org/10.1172/JCI160517

Zhu, P., Wu, J., Wang, Y., Zhu, X., Lu, T., Liu, B., He, Luyun, Ye, B., Wang, S., Meng, S., Fan, D., Wang, J., Yang, L., Qin, X., Du, Y., Li, C., He, Lei, Ren, W., Wu, X., Tian, Y., Fan, Z., 2018. LncGata6 maintains stemness of intestinal stem cells and promotes intestinal tumorigenesis. Nat. Cell Biol. 20, 1134–1144. https://doi.org/10.1038/s41556-018-0194-0


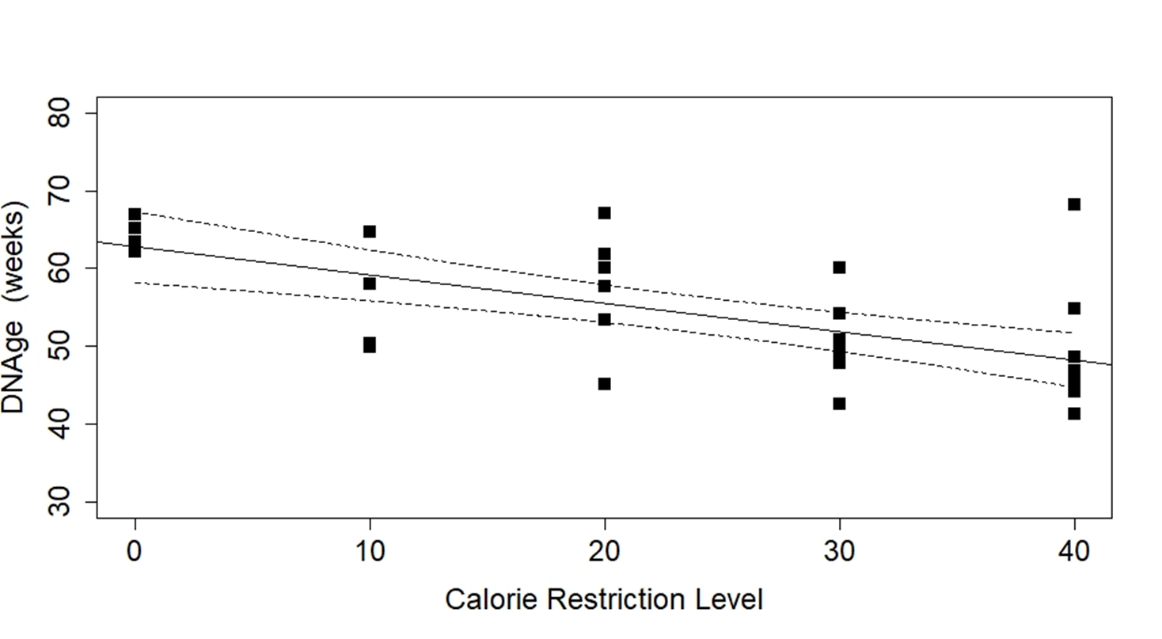


**Supplementary Material Figure 1**. DNAge® (an algorithmically estimated DNA methylation age) by graded calorie restriction (CR) level for 24-month-old (104 weeks) male C57BL/6J mice following 19 months CR. Linear regression of DNAm against CR (solid line) was significant, F_1, 30_ = 20.71, p < 0.0001 after removal of one outlier from the 30CR group ( a mouse which was determined by necropsy to have liver neoplasia, it accounted for 42% of all DNAm outliers and had a DNAge® of 131 weeks). Confidence intervals are 95% (dashed lines).

**
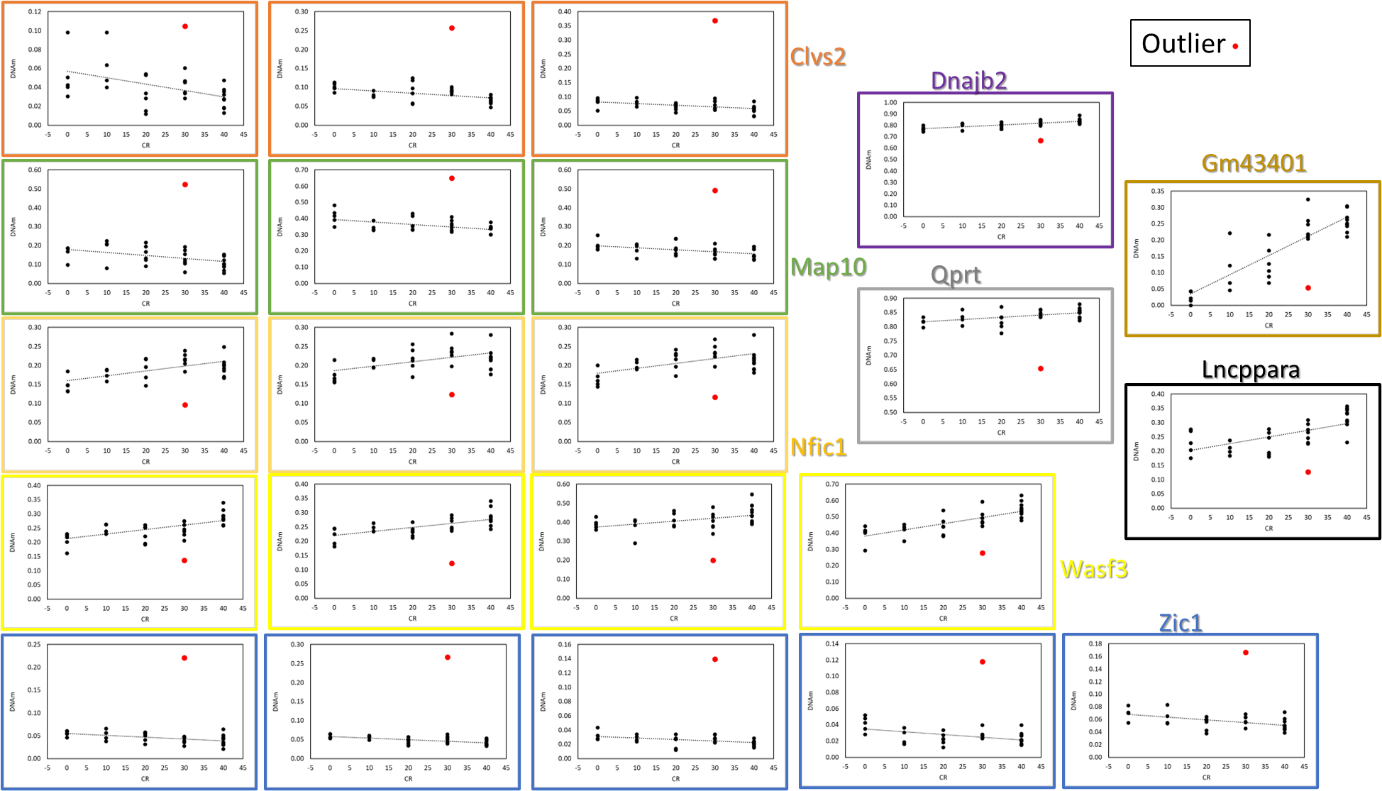
**

**Supplementary Material Figure 2**. DNAm vs CR at significant CpG sites illustrated in main text Figure 2 at which a mouse with liver neoplasia was identified to have an outlier DNAm value. In all cases the outlier is above the regression line the gradient is negative and below the line if the gradient is positive. Based on our observations in these CpG sites of epigenetic rejuvenation with increasing CR the outliers lie in the direction of a mouse of extreme old age.
